# Rare‐Earth Mediated Engineering in ZnSe@ZnS:Eu^3^^+^ to Simultaneously Achieve Structural Modulation and Atomic Eu Doping Sites for X‐Ray Imaging

**DOI:** 10.1002/advs.75871

**Published:** 2026-05-29

**Authors:** Jicun Ma, Chenhao Yang, Jiada Fan, Haorong Jiao, Jiawang Liu, Jialiang Xu, Hui Cai, Yinghui Wang, Boyuan Shen, Jiabin Cui

**Affiliations:** ^1^ State Key Laboratory of Radiation Medicine and Protection School of Radiation Medicine and Protection Collaborative Innovation Center of Radiological Medicine of Jiangsu Higher Education Institutions Soochow University Suzhou P. R. China; ^2^ Femtosecond Laser Laboratory College of Physics Synergetic Extreme Condition High‐Pressure Science Center Jilin University Changchun P. R. China; ^3^ State Key Laboratory of Bioinspired Interfacial Materials Science Institute of Functional Nano & Soft Materials (FUNSOM) Soochow University Suzhou Jiangsu P. R. China

**Keywords:** atomic doping sites, coupled semiconductor, rare‐earth doping, scintillators, X‐ray imaging

## Abstract

Lanthanide ions offer distinctive optical features but have weak absorption due to parity‐forbidden 4f‐4f transitions, prompting strategies to boost absorption through host matrices, dopant engineering, and optimized nanostructures. Ln‐doped colloidal QDs enhance photoluminescence and broaden absorption for applications in solar energy and photo‐catalysis, yet integrating Ln^3+^ into hosts and achieving efficient energy transfer, especially in Ln‐doped II‐VI sulfide QDs, still remains challenging and unsolved. Here, we report a “dual hard‐base anchoring” strategy for synthesizing rare‐earth doped ZnSe@ZnS:Eu^3+^ QDs. Interestingly, based on the fluoride‐oleylamine synthetic interactions, Eu^3+^ forms atomic Eu doping sites on the QD surface, as shown by extended X‐ray absorption fine‐structure analysis. Optimized temperature, Eu^3+^ doping, and F^−^ concentration yield controlled morphologies, including tetragonal growth, tip, island, and coupled flower forms. Surface passivation with oleylamine and a 1,10‐phenanthroline ligand broadens Eu^3+^‐related emission (^5^D_0_→^7^F_2_/^7^F_1_). The approach also applies to six other rare‐earth dopants, delivering high performance in X‐ray imaging and enabling rare‐earth related new applications.

## Graphical abstract

A dual hard‐base anchoring strategy was developed to synthesize ZnSe@ZnS:Eu^3+^ quantum dots, leveraging fluoride‐oleylamine interactions to create surface atomic Eu doping sites. By precisely tuning temperature, Eu^3+^ concentration, and fluoride levels, we obtain morphologies ranging from tip growth to coupled flower‐like structures. Passivation with oleylamine (OAm) and 1,10‐phenanthroline (Phe) not only enhances Eu^3+^‐related emission but also enables their use as X‐ray scintillators.

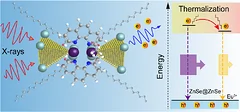

## 1 Introduction

Lanthanide ions, celebrated for their abundant 4f electrons, are widely exploited in optical applications due to their distinctive electronic structures [1, 2, 3, 4, 5, 6, 7]. Yet their performance is constrained by the parity‐forbidden nature of 4f‐4f transitions, which yields narrow emission features but inherently weak absorption and limited photo‐conversion efficiency, ultimately leading to limited quantum yields [1, 4, 8]. This limitation has spurred efforts to elevate the absorption cross‐section of lanthanide centers [9, 10]. Toward this end, strategies include employing host matrices that promote efficient energy transfer, dopant engineering to introduce advantageous energy levels, and designing nanostructures that optimize light‐matter coupling [8, 11, 12, 13].

Lanthanide‐doped colloidal quantum dots (Ln‐doped QDs) represent a significant step forward, extending the capabilities of conventional QDs while markedly enhancing the photoluminescence efficiency of lanthanide centers [3, 8, 14, 15, 16]. Not only do these systems enable precise control over size, composition, and morphology for tailor optical proper [17], but they also exhibit substantial absorption cross‐sections, unlocking diverse applications in solar energy, photo‐catalysis, and beyond. This progress naturally raises the prospect of synergistically integrating the advantageous attributes of lanthanide ions with QDs to realize Ln‐doped QDs that showcase their distinctive optical features and broad application potential [18, 19, 20].

In parallel, lanthanides ions, as hard acids, exhibit a strong affinity for hard bases such as PO_4_^3^^−^, OH^−^, CH_3_COO^−^, and F^−^, reflecting high oxophilicity and relatively low thiophilicity [21, 22, 23, 24, 25]. Consequently, Ln‐doped perovskites have been successfully synthesized, with promising demonstrations of functional utility [11, 14, 26, 27]. However, owing to the weak stability and susceptibility to moisture and oxygen of Ln‐CsPbBr_3_, the widely application of the Ln‐CsPbBr_3_ remains limited [28, 29]. Moreover, the trivalent Ln^3+^ state complicates their incorporation into host matrices, particularly due to lattice‐mismatch with II‐VI sulfide QDs [1, 18, 30, 31]. Despite the potential to engineer Ln‐modulated II‐VI sulfide QDs, progress in this area remains limited, hindered by challenges in achieving optimal doping levels, preserving structural integrity, and ensuring efficient energy transfer between lanthanide ions and the host lattice [15, 24, 32, 33].

**FIGURE 1:**
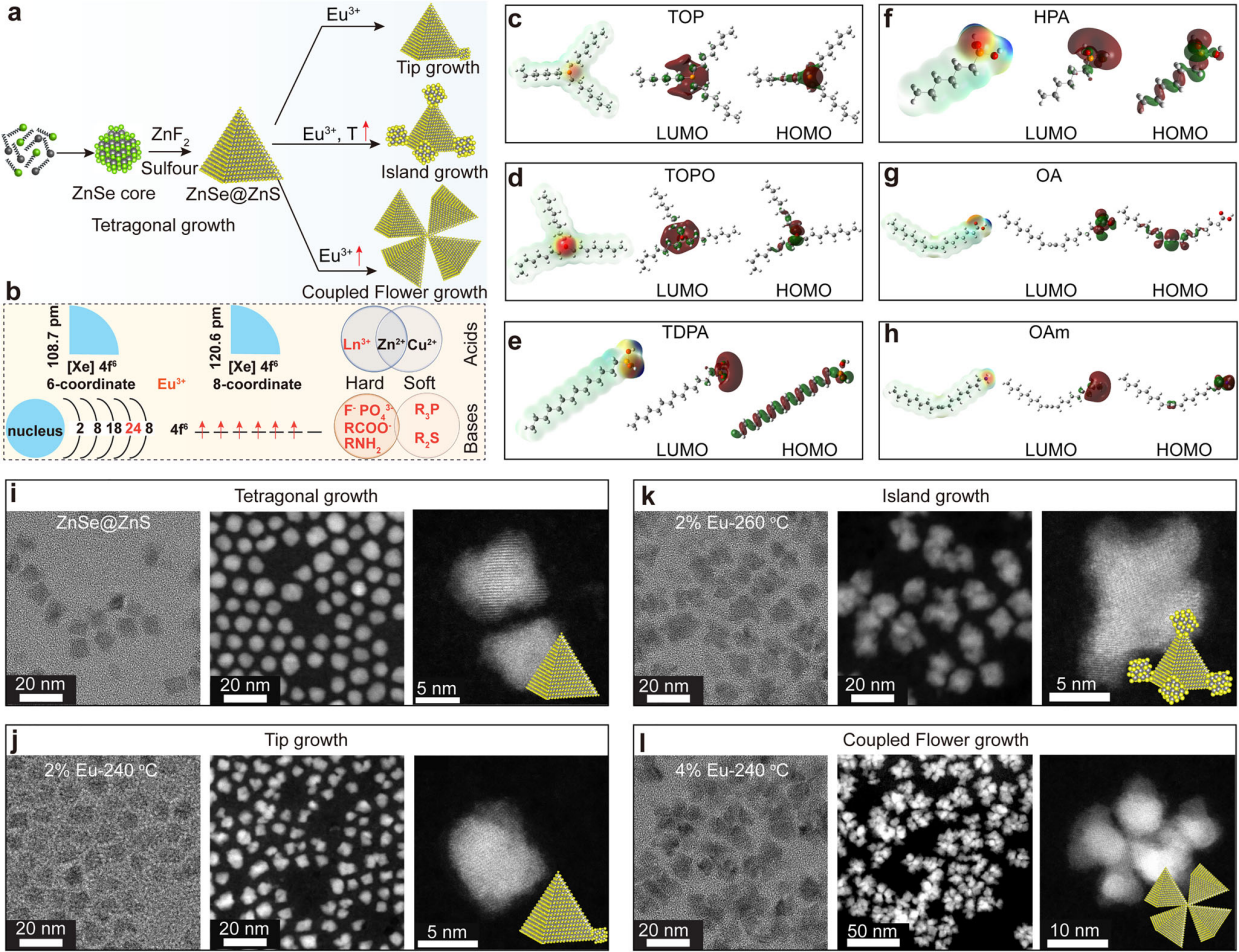
Rare‐earth ions mediated ZnSe@ZnS:Eu^3+^ morphology manipulation strategy. (a) The cartoon for the Eu^3+^/F^−^ ions modulated morphologies of ZnSe@ZnS:Eu^3+^ QDs from tetragonal tip growth, island growth to coupled flower growth. (b) The electron configuration, radius, and coordinate number of Eu^3+^ ions. The inserts reflect the standard hard‐soft acid‐base (HSAB) theory, particularly for rare‐earth ions and ligands. The calculated electrostatic potential and dipolar moment, highest occupied molecular orbital (HOMO) and lowest occupied molecular orbital (LUMO) molecular orbital energy level of triphenylphosphine (TOP, c), trioctylphosphine oxide (TOPO, d), tetradecylphosphonic acid (TDPA, e), n‐hexylphosphonic acid (HPA, f), oleic acid (OA, g), and oleylamine (OAm, h) of typical ligands based on the HSAB theory. The transmission electron microscopy (TEM) imaging and scanning transmission electron microscope (STEM) imaging under low magnification, and STEM under high magnification of tetragonal ZnSe@ZnS QDs (i), tetragonal tip growth (j), tetragonal island growth (k), and tetragonal coupled flower growth (l) ZnSe@ZnS:Eu^3+^ QDs, respectively.

To address these challenges, we developed a “*dual hard‐base anchoring*” strategy for the successful synthesis of rare‐earth doped II‐VI ZnSe@ZnS:Eu^3+^ QDs (Figure 1a). Based on the hard‐soft acid‐base (HSAB) theory and leveraging interactions between fluoride ions and oleylamine (OAm), Eu^3+^ was stabilized and immobilized to form atomic Eu doping sites on the surface of ZnSe@ZnS QDs, as identified by extended X‐ray absorption fine‐structure (EXAFS) analysis. With optimizing the temperature, Eu^3+^ doping concentration, and F^−^ ion content, we achieved precise morphology control, yielding tetragonal growth, tetragonal tip growth, tetragonal island growth, and tetragonal coupled flower‐like growth of ZnSe@ZnS:Eu^3+^ QDs. Furthermore, surface passivation with the synergistic effects of OAm and 1,10‐phenanthroline (Phe) ligands led to a significantly broadening of the Eu^3+^‐related (^5^D_0_→^7^F_2_ and ^5^D_0_→^7^F_1_ transitions) photoluminescence spectrum. This strategy was also applicable to other rare‐earth doped ZnSe@ZnS QDs, such as the La^3+^, Ce^3+^, Pr^3+^, Sm^3+^, Tb^3+,^ and Yb^3+^ ions dopants. Finally, the precisely controlled ZnSe@ZnS:Eu^3+^ QDs were successfully employed as X‐ray scintillators, opening new avenues for rare‐earth doped QDs enabled applications in photosensing, energy conversion, and security.

## 2 Results and Discussion

### 2.1 Synthesis and Characterization of ZnSe@ZnS:Eu^3+^ QDs

An overview of the synthesis of ZnSe@ZnS:Eu^3+^ QDs with rare‐earth ion mediated morphology engineering is shown in Figure 1a. Within the framework of HSAB theory (Figure 1b), Zn^2+^ functions as an intermediate acid with balanced oxophilicity and thiophilicity. Accordingly, all syntheses were conducted under rigorously anhydrous and oxygen‐free conditions. In contrast, rare‐earth ions behave as typical hard acids with a large ionic radius, exhibiting weak affinity with sulfur (a soft base) but strong affinity for hard bases such as PO_4_^3^^−^, NH_2_‐R, CH_3_COO^−^, and F^−^. To elucidate the factors governing morphological evolution, we systematically screened a series of ligands, starting with the soft‐base triphenylphosphine (TOP). As illustrated in Figure 1c, the electrostatic potential, dipole moment, and the highest occupied molecular orbital (HOMO) and lowest unoccupied molecular orbital (LUMO) energy levels of TOP were computed to assess its influence on the morphology of ZnSe@ZnS:Eu^3+^ QDs. Corresponding TEM and STEM images of TOP‐mediated structures are provided in Supplementary Figure S1. We further investigated a panel of hard‐base ligands (Figure 1d–h), including trioctylphosphine oxide (TOPO; Figure 1d), tetradecylphosphonic acid (TDPA; Figure 1e), n‐hexylphosphonic acid (HPA; Figure 1f), oleic acid (OA; Figure 1g), and oleylamine (OAm; Figure 1h) for the fabrication of ZnSe@ZnS QDs.

Upon introduction of the ZnF_2_ precursor, variations in the amount of F^−^ (from 0.2 mL to 0.5 mL) lead to distinct morphological outcomes (Supplementary Figure S2). At approximately 0.4 mL of ZnF_2_, monodispersed single tetragonal ZnSe@ZnS QDs are obtained. In contrast, either lower or higher amounts of ZnF_2_ precursor result in the formation of coupled ZnSe@ZnS dimer QDs. These observations indicate that F^−^ not only passivates surface defects and enhances stability but also immobilizes surface‐doped lanthanide ions, thereby modulating the morphology. Notably, ZnSe@ZnS QDs without lanthanide ions doping exhibit typical tetragonal growth (Figure 1i). With the addition of Eu^3+^ ions, a pronounced tetragonal tip growth morphology emerges (Figure 1j), attributed to the high activity of the tip regions due to strong bonding with fluoride ions. At the same Eu^3+^ doping level, a tetragonal island growth mode is also observed (Figure 1k, Supplementary Figure S3), which may result from high‐temperature enhanced ion diffusion coupled with lattice mismatch between ZnSe and ZnS, leading to a transition from epitaxial growth to irregular island growth. Furthermore, as the Eu^3+^ doping level increases from 2% to 4%, the markedly enhanced binding affinity between F^−^ and Eu^3+^ promotes a tetragonal coupled flower growth morphology (Figure 1l, Supplementary Figures S4 and S5, and Supplementary Table S1) in ZnSe@ZnS:Eu^3+^ QDs. Additional morphological modulation mediated by TDPA and TOPO ligands under optimized Eu^3^^+^ doping is illustrated in Supplementary Figures S6–S8. Thus, by appropriate surface ligands and controlled rare‐earth ion incorporation, we achieve both high‐radius lanthanide doping and precise morphology tuning in Eu^3^^+^‐doped ZnSe@ZnS QDs.

### 2.2 Structural Characterization of ZnSe@ZnS:Eu^3+^ QDs

**FIGURE 2:**
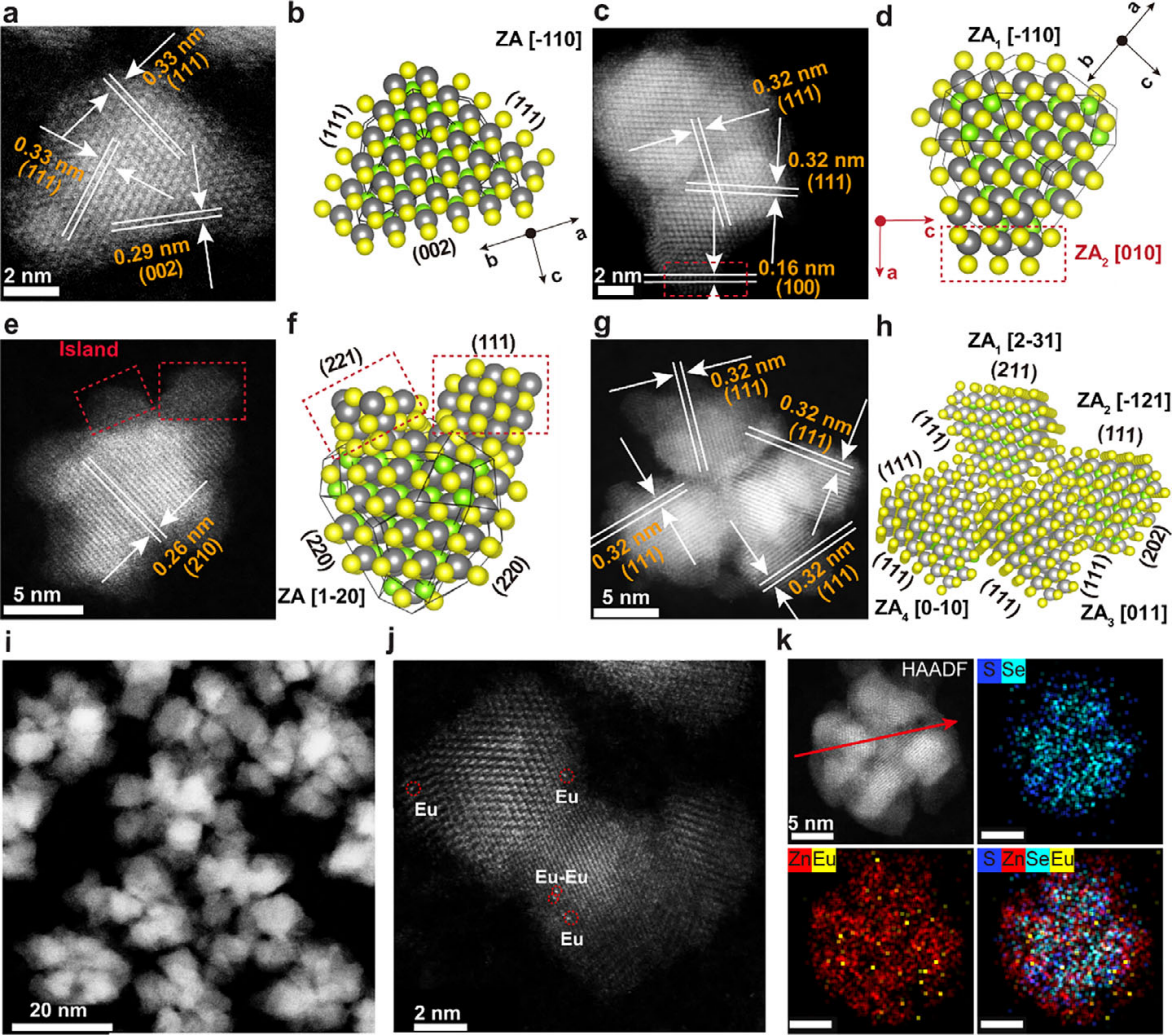
Structural analysis of different growth types of ZnSe@ZnS:Eu^3+^ QDs. The high‐angle annular dark field scanning transmission electron microscope (HAADF‐STEM) imaging (a, c, e, and g) and the corresponding 3D reconstructed models (b, d, f, and h) of Tetragonal growth (a, b), Tetragonal tip growth (c, d), Tetragonal island growth (e, f), and Tetragonal coupled flower growth (g, h) ZnSe@ZnS:Eu^3+^ QDs. The STEM imaging (i), high resolution STEM imaging (j), and an energy dispersive X‐ray spectroscopy (EDS) detector (EDS‐STEM) mapping (k) of tetragonal coupled flower growth ZnSe@ZnS:Eu^3+^ QDs.

To further analyze the aforementioned growth modes, we performed high angle annular dark field STEM (HAADF‐STEM) imaging for detailed structural characterization. As depicted in Figure 2a, the low‐index lattice planes (111) and (002) of the typical zinc blende (ZB) structure (Supplementary Figure S9) were observed along the zone axis (ZA) [$\bar{1}$10], consistent with the simulated 3D atomic model of ZnSe@ZnS (Figure 2b). HAADF‐STEM imaging (Supplementary Figure S9) further confirmed the successful synthesis of tetragonal ZnSe@ZnS QDs. The core‐shell element distribution was further identified by energy‐dispersive X‐ray spectroscopy (EDS) line scans and X‐ray Diffraction (XRD, Supplementary Figure S10), showing selenium concentrated in the core and sulfur distributed predominantly in the shell. Fine‐structure analysis of tetragonal tip growth ZnSe@ZnS:Eu^3+^ QDs (Figure 2c,d) presented roughening at the tip region, along with the observation of a small ZnS domain viewed along the ZA [010]. EDS mapping and line scans confirmed the incorporation of europium (Supplementary Figure S11). In comparison, the tetragonal island growth ZnSe@ZnS:Eu^3+^ QDs exhibited a significant increase in small ZnS domains (Figure 2e,f, Supplementary Figure S12). Meanwhile, the tetragonal island growth QDs exhibit a “star‐like” morphology. When the Eu^3+^ concentration was increased to 4%, the strong affinity among Eu^3+^, F^−^, and surface ligands promoted the formation of a tetragonal coupled “flower‐like” architecture, wherein multiple ZnSe@ZnS:Eu^3+^ QDs adherent together. As depicted in Figure 2g,h, four ZB ZnSe@ZnS:Eu^3+^ QDs couple under ZA_1_ [$2\bar{3}1$], ZA_2_ [$\bar{1}21$], ZA_3_ [011], and ZA_4_ [$0\bar{1}0$], respectively. The detailed tetragonal coupled flower growth characterization, including STEM, HAADF‐STEM, and EDS element mapping presented in Figure 2i–k, demonstrates the coexistence of both isolated single‐atom Eu sites and adjacent Eu‐Eu dual‐atom sites within the imaged regions, with no evidence of larger Eu clusters. To further elucidate the impact of Eu doping on the ZnSe@ZnS matrix, supplementary XRD analysis (Supplementary Figure S10) reveals a slight shift of the (111), (220), and (311) diffraction peaks toward lower angles after Eu^3+^ incorporation, consistent with lattice expansion attributable to the larger ionic radius of Eu^3+^ relative to Zn^2+^. Additionally, peak broadening indicates defect formation and minor lattice distortion induced by Eu^3+^ doping. Collectively, these observations provide direct evidence for atomic Eu incorporation into ZnSe@ZnS, yielding ZnSe@ZnS:Eu^3+^ quantum dots.

Moreover, the detailed femtosecond transient absorption (TA) maps for different growth features were depicted in Supplementary Figure S13. As seen in Supplementary Figure S13a, a broad spectral feature located at 430.0 nm appears in the TA spectra of QDs without doping. In addition, it is interesting to find that a small negative signal located at 455.0 nm exists in the TA spectra (Supplementary Figures S13e), which was assigned to the simulated emission (SE) manifesting the possibility of the formation of the surface trapping sites in the QDs. After the formation of tetragonal tip growth, the corresponding TA map is similar with that without doping. The only difference is the enhancement of SE located at 455.0 nm, since the introduction of Eu^3+^ maybe creates many trapping‐sites, which facilitate the photoluminescence originated from trapping‐sites. After the temperature increases from 240°C to 260°C with the formation of tetragonal island growth of QDs, this SE signal keeps enhancement as seen in Supplementary Figure S13b,c and Supplementary Figure S13f,g. This suggests that many carriers jump into trapping sites through thermal activation, causing the enhancement of simulated emission. If the amount of Eu^3+^ increases from 2% to 4% with the formation of tetragonal coupled flower growth, trapping‐sites may keep increasing (Supplementary Figure S13d and Supplementary Figure S13h). This causes many photo‐generated carriers jumps into trapping‐sites, leading to the enhancement of simulated emission at 455.0 nm. In a word, increasing the temperature or increasing the percentage of Eu^3+^ ion can both facilitate the trapping sites participating the photoluminescence of QDs. These results demonstrate that the morphology of ZnSe@ZnS:Eu^3+^ QDs can be effectively tailored through precise control of Eu^3^^+^ concentration, reaction temperature, and fluoride precursor amount.

### 2.3 Structural Simulation and Formation Mechanism Study

**FIGURE 3:**
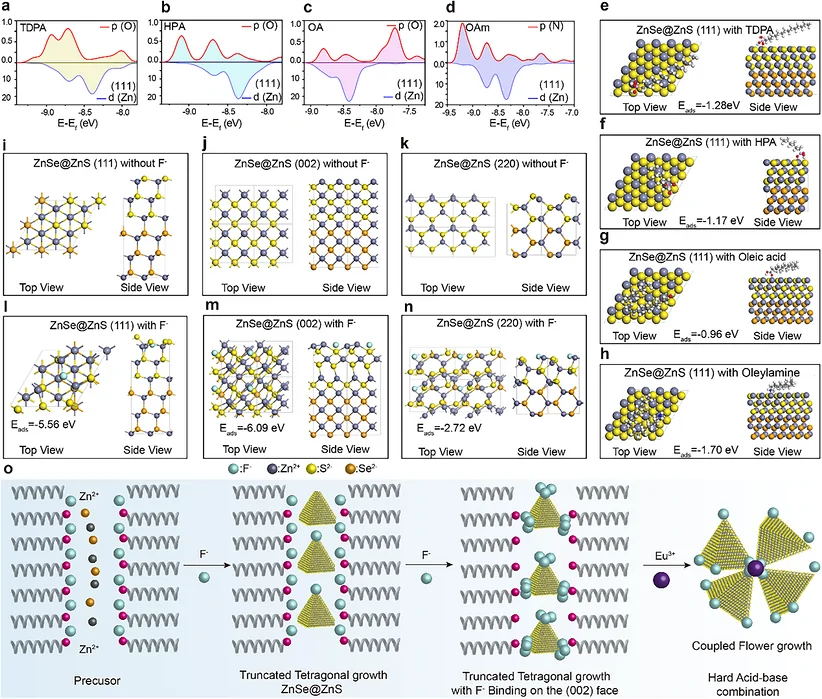
Ligands effect and calculation study based on HSAB theory for ZnSe@ZnS:Eu^3+^ QDs. The partial density of states (pDOS, a‐d) and adsorption energy calculation (e‐f) of (111) lattice plane for ZnSe@ZnS QDs with TDPA (a, e), HPA (b, f), OA (c, g), and OAm (d, h), respectively. The calculated adsorption energy of (111), (002), and (220) lattice plane for ZnSe@ZnS QDs without (i‐k) and with (l‐n) F^−^ ions effect. The proposed growth mechanism (o) for F^−^ ions and Eu^3+^ mediated morphology modulation of tetragonal coupled flower coupled growth ZnSe@ZnS:Eu^3+^ QDs.

To fully understand the mechanism for the Eu^3+^ doped ZnSe@ZnS QDs growth, the theoretical simulation was investigated. The partial density of states (pDOS) analysis revealed hybridization between the *d‐* orbital of Zn atoms and the *p‐* orbital of N atoms in OAm or O atoms in HPA, TDPA, and OA on the (111) plane of ZnSe@ZnS QDs was depicted in Figures 3a‐d and Supplementary Figure S14. Specifically, when OAm is adsorbed on the (111) plane, the *p‐d* hybridization peaks appear between ‐8.0 and ‐9.4 eV, which were significantly lower than that of HPA, TDPA, and OA. And the absorption energy of TDPA, HPA, OA, and OAm (Figure 3e–h, Supplementary Table S2) on the (111) plane of ZnSe@ZnS QDs was ‐1.28, ‐1.17, ‐0.96, and ‐1.70 eV, respective. That is the typical OAm presents high affinity for Zinc. Additionally, the F^−^ affected adsorption calculation was also taken into consideration. As depicted in the Figure 3i–n, it was clearly found that the absorption energy of F^−^ on the ZnSe@ZnS QDs presents a significant difference. And the absorption energy on the (002) plane was lowest reached to ‐6.09 eV comparing that under (111), and (220) plane of ZnSe@ZnS QDs (Supplementary Table S3). That is, with the synergetic effect of OAm and F^−^ ligands, the truncated tetragonal ZnSe@ZnS QDs were fabricated. Then, with the high absorption energy of F^−^ on the (002) plane and also the high activity of tip location with three dangling bonds, when the Eu^3+^ introduced in the system, owing to the high affinity of F^−^ and Eu^3+^ based on the HSAB theory, results in the formation of tetragonal coupled flower growth (Figure 3o) of ZnSe@ZnS:Eu^3+^ QDs [13, 23]. Thus, the suitable optimized parameters play significant roles in the morphology of ZnSe@ZnS:Eu^3+^ QDs.

### 2.4 Optical Signatures for ZnSe@ZnS:Eu^3+^ QDs

**FIGURE 4:**
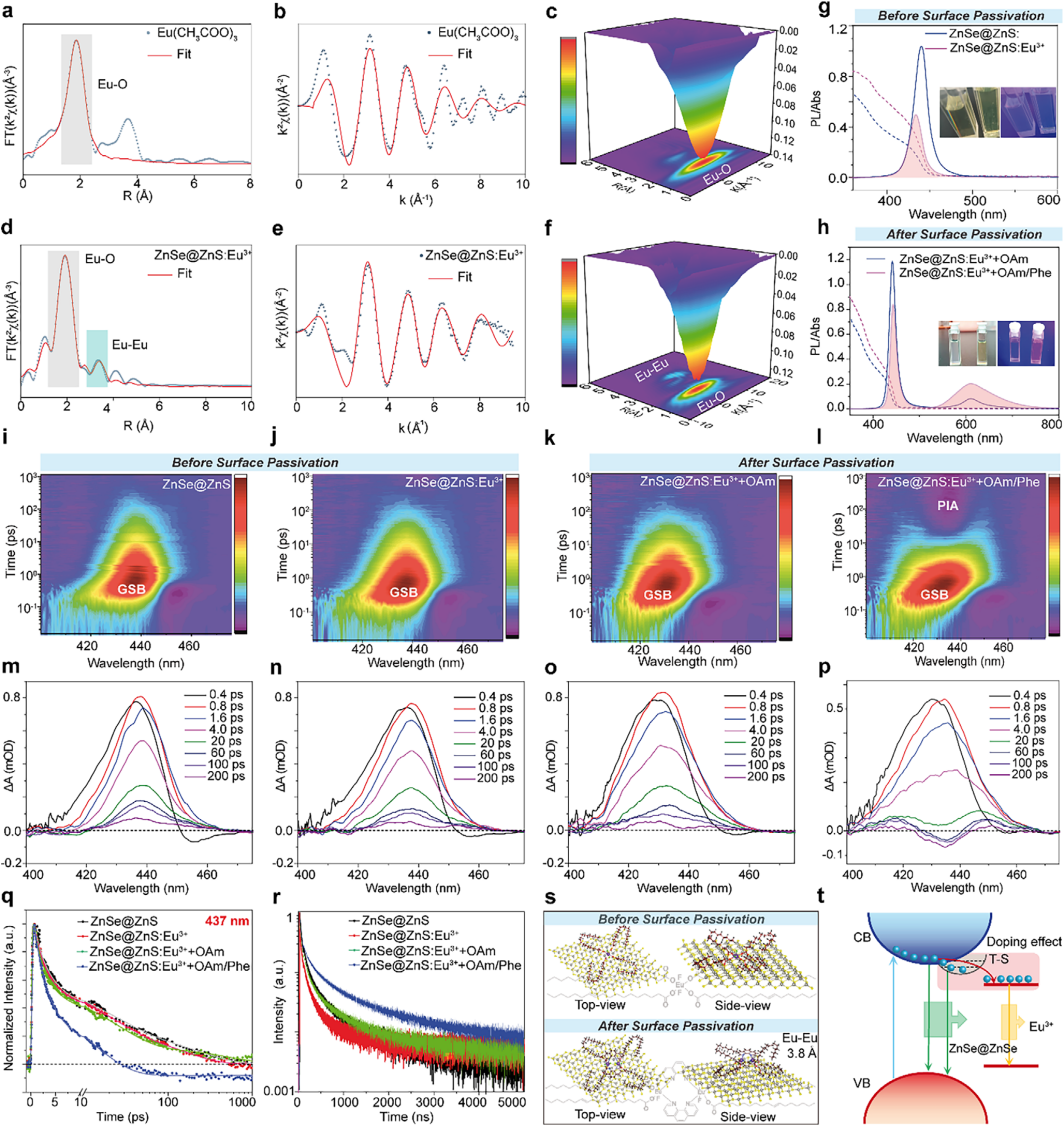
Spectrum study for Eu doping effect of ZnSe@ZnS:Eu^3+^ QDs. The extended X‐ray absorption fine structure (EXAFS) in R‐space (a, d), k‐space (b, d), and Counter plot Morlet (4, 1) WT amplitude spectrum of standard Eu(CH_3_COO)_3_ sample (a‐c) and ZnSe@ZnS:Eu^3+^ (d‐f) QDs, respective. The absorption and photoluminescence spectrum of ZnSe@ZnS and ZnSe@ZnS:Eu^3+^ QDs before (g) and after (h) surface passivation. The 2D transient absorption (TA) surface plot (i‐l) and corresponding decay TA spectroscopy (m‐p) with different probe delay times under 365 nm excitation of ZnSe@ZnS (i, m), ZnSe@ZnS:Eu^3+^ (j, n), ZnSe@ZnS:Eu^3+^ with OAm passivation (k, o), and ZnSe@ZnS:Eu^3+^ with OAm/Phe passivation (l, p), respectively. The normalized decay kinetic curves analysis under 430.0 nm (q) and photoluminescence lifetime decay curves (r) of related QDs, respective. (s) The proposed atomic structure model before and after surface passivation for the “Eu‐Eu” dual‐atom sites with 3.8 Å. (t) The proposed photo‐carriers transition mechanism for ZnSe@ZnS:Eu^3+^ QDs.

To further validate the interfacial bonding between Eu^3+^ ions and ZnSe@ZnS QDs, we conducted extended X‐ray absorption fine structure (EXAFS) measurements on Eu^3+^ within Eu(CH_3_COO)_3_ and ZnSe@ZnS:Eu^3+^ QDs. The Eu L_3_‐edge X‐ray absorption near‐edge structure (XANES) spectra (Supplementary Figure S15) display a characteristic white‐line peak in both Eu(CH_3_COO)_3_ and ZnSe@ZnS:Eu^3+^, indicating Eu in the +3 oxidation state (Eu^3+^). To gain insight into the local coordination around Eu^3+^ ions, first‐shell near‐neighbor (NN) fitting in R‐space, k‐space, and Counter plot Morlet (4, 1) WT amplitude spectrum of Eu(CH_3_COO)_3_ and ZnSe@ZnS:Eu^3+^ were performed on the Fourier‐transform EXAFS data (Figure 4a–f, Supplementary Table S4). In Eu(CH_3_COO)_3_, Eu‐O single‐scattering paths (R ≈ 1.99 ± 0.01 Å) dominate, consistent with a typical coordination number (CN) of ∼6. In ZnSe@ZnS:Eu^3+^, the first‐shell region broadens around ≈1.9 Å, indicating contributions from multiple scattering species (e.g., O and F). The fitting yields Eu‐O at 1.99 ± 0.08 Å and Eu‐F at 2.31 ± 0.09 Å, in good agreement with theoretical expectations (Eu‐O ≈ 1.99 Å; Eu‐F ≈ 2.31 Å). Moreover, the second shell reveals an Eu‐Eu interaction with CN ≈ 1.05 and R ≈ 3.76 Å, and together with near‐unity Eu‐Eu CN, manifesting the extensive Eu dual‐atom clustering. These results indicate that Eu^3+^ exists as Eu‐O and Eu‐F coordinated Eu^3+^ species, with Eu‐Eu dual‐atom domains acting as the structural units stabilized by ZnSe@ZnS matrix. Collectively, the XANES analysis confirms Eu^3+^ incorporation into ZnSe@ZnS QDs, with Eu^3+^‐Eu^3+^ dual‐atom domains embedded in a mixed O/F ligands coordination environment.

The absorption and photoluminescence (PL) spectra of ZnSe@ZnS and ZnSe@ZnS:Eu^3+^ QDs are shown in Figure 4g, with an absorption peak at ∼435.0 nm and a PL peak at 440.0 nm. After the doping of Eu^3+^ into ZnSe@ZnS QDs, the absorption peak blue‐shifts to 423.2 nm and the PL peak to 436.0 nm, while the PL intensity decreases due to Eu^3+^‐induced trapping sites that create a competing non‐radiative channel. The photoluminescence quantum yield (PLQY) of ZnSe@ZnS and ZnSe@ZnS:Eu^3+^ was determined to be approximately 13.5% and 3.7%, respectively (Supplementary Table S5). This observation indicates the practical existence of nonradiative pathways introduced by Eu^3+^ incorporation, which reduces the PL quantum efficiency from 13.5% to 3.7%. To optimize ZnSe@ZnS:Eu^3+^ PL, surface‐passivating ligands (OAm and Phe) were employed to passivate defect sites on the QDs (Figure 4h). In the presence of OAm/Phe, the intrinsic ZnSe@ZnS emission at 440.0 nm diminishes while Eu^3+^ emission at 610.0 nm rises, demonstrating that enhanced surface passivation suppresses nonradiative channels and simultaneously facilitates energy transfer from the QDs to Eu^3+^ ions. The ligands also yield a measured PLQY of about 23.1%, confirming that OAm and Phe appreciably improve the PL properties of ZnSe@ZnS:Eu^3+^.

The femtosecond transient absorption (TA) maps for ZnSe@ZnS, ZnSe@ZnS:Eu^3+^, ZnSe@ZnS:Eu^3+^ with OAm, and ZnSe@ZnS:Eu^3+^ with OAm/Phe treatment are shown in Figure 4i–l, and their corresponding time‐resolved spectra are given in Figure 4m–p at the same time. After photo‐excitation, a broad positive signal at 435.0 nm appears in these TA maps, which should be attributed to ground‐state bleaching (GSB) and stimulated emission (SE) according to the steady absorption and PL spectra in Figure 4g,h. Its amplitude gradually weakens with delay time, reflecting the photo‐generated carriers gradually decay from the initial conduction band to the valence band. However, a new transient species, corresponding to photo‐induced absorption (PIA), gradually appears in the TA map of ZnSe@ZnS:Eu^3+^ with OAm/Phe treatment, showing a negative signal with a long lifetime exists in the time‐resolved spectra after ∼30 ps as seen in Figure 4l. This suggests a new intermediate state with a long lifetime is formed after photoexcitation. The initial ∼1.0 ns dynamics are similar for ZnSe@ZnS, ZnSe@ZnS:Eu^3+^, and ZnSe@ZnS:Eu^3+^ with OAm, but ZnSe@ZnS:Eu^3+^ with OAm/Phe shows a negative PIA peak at ∼30.0 ps that persists to 1 ns, consistent with a Eu^3+^‐related emission state. As depicted in Figure 4q, the GSB curves of ZnSe@ZnS, ZnSe@ZnS:Eu^3+^, and ZnSe@ZnS:Eu^3+^ with OAm display similar relaxation behavior within ∼1 ns, which is almost consistent with those in TA map and time‐resolved spectra. But the TA curves of ZnSe@ZnS:Eu^3+^ with OAm/Phe treatment show an unconventional relaxation behavior, exhibiting the GSB signal decays rapidly and then transist to PIA located at 435.0 nm, corresponding to the emission state. This should be responsible for the fact the ligands OAm and Phe promote the PLQY of ZnSe@ZnS:Eu^3+^ increases from 3.7% to 23.1%.

The photoluminescence lifetime decays were depicted in Figure 4r and Supplementary Figure S16. When OAm is added to ZnSe@ZnS:Eu^3+^ QDs, the fluorescence relaxation becomes slower, reflecting passivation of surface defects by OAm. Additionally, ZnSe@ZnS:Eu^3+^ with OAm/Phe exhibits even slower transient relaxation than ZnSe@ZnS:Eu^3+^ with OAm, indicating that Phe not only passivates surface defects but also stabilizes and immobilizes the Eu‐Eu dual‐atom sites (Figure 4s), facilitating the formation of intermediate state in ZnSe@ZnS:Eu^3+^ with OAm/Phe. This intermediate state can enhance the energy transfer from ZnSe@ZnS to Eu^3+^ after photo‐excitation, inducing the emission of Eu^3+^ in Figure 4h. The emission mechanism occurring in the QDs can be illuminated in Figure 4t. Eu^3+^ can cause defects that create non‐radiative pathways and accelerate relaxation, while Phe and OAm stabilize Eu^3+^ and promote energy transfer from the QD to Eu^3+^, yielding enhanced, broad emission characteristic of Eu^3+^‐doped QDs and manifesting potential applications for earth‐rare ion‐doped II‐VI QDs.

### 2.5 X‐Ray Imaging Application Study

**FIGURE 5:**
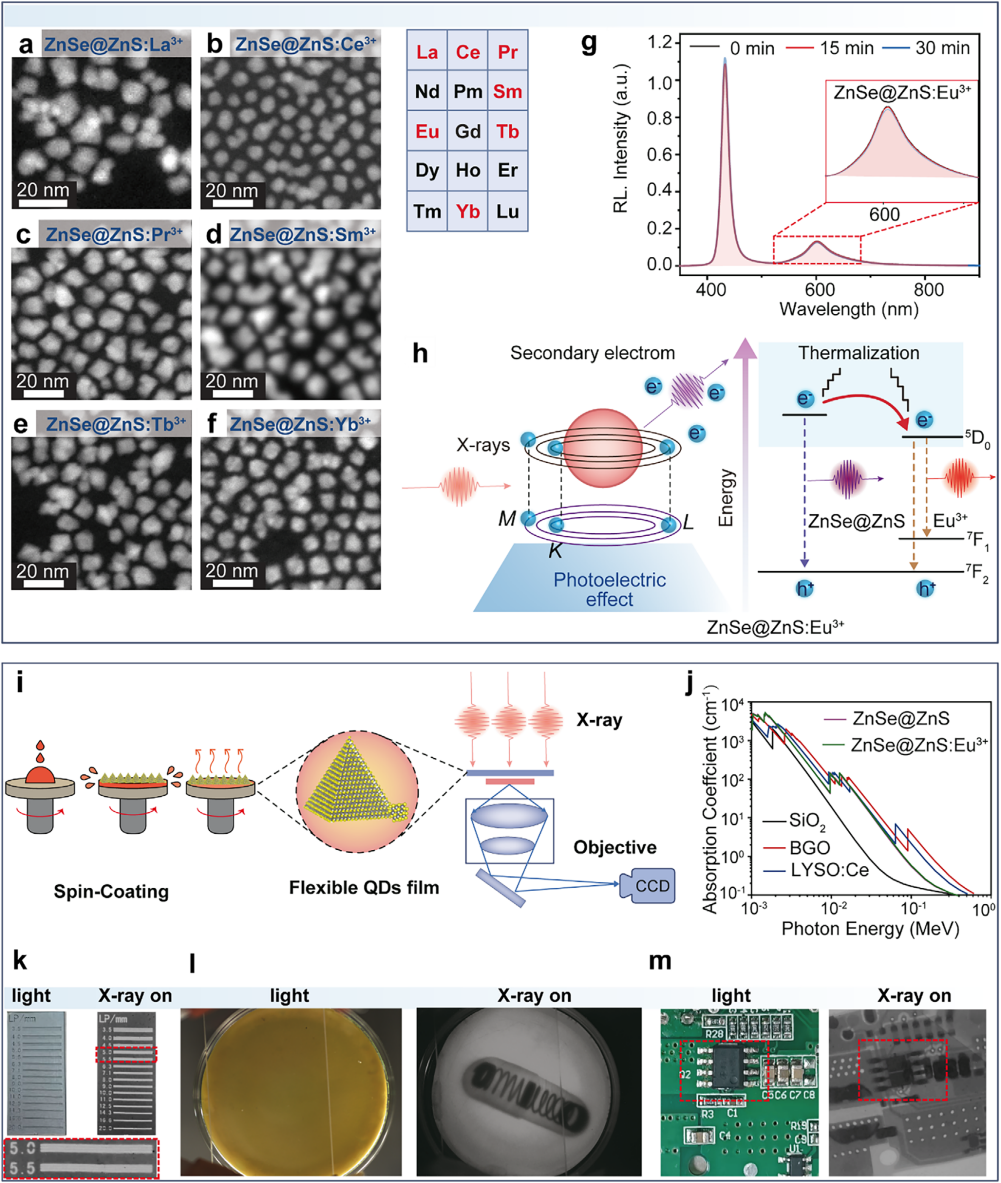
X‐ray imaging and scintillator properties application. The STEM imaging of La^3+^ (a), Ce^3+^ (b), Pr^3+^ (c), Sm^3+^ (d), Tb^3+^ (e), and Yb^3+^ (f) ions modulated ZnSe@ZnS:R QDs, respectively. The X‐ray radio‐luminescence spectrum with different exposure time (g), X‐ray radiation induced photo‐carriers transition mechanism (h), flexible QDs film fabrication for X‐ray imaging (i), Absorption coefficient (j) and related X‐ray imaging (k‐m) of ZnSe@ZnS and ZnSe@ZnS:Eu^3+^ QDs.

To further explore the general strategy for the other rare‐earth doped ZnSe@ZnS QDs, the La^3+^, Ce^3+^, Pr^3+^, Sm^3+^, Tb^3+^ and Yb^3+^ ions doped QDs were synthesized, and their morphology and optical properties were shown in Figures 5a‐f and Supplementary Figures S17 and S18. The TA spectroscopy of ZnSe@ZnS QDs doped with different Ln(III) ions were displayed in Supplementary Figure S19. It seemed that rare earth ions with smaller sizes are more prone to forming trapping sites on QDs. This demonstrates that our “*dual hard‐base anchoring strategy*” can not only modulate morphology but also broaden the possibilities for rare‐earth ion doping in II‐VI QDs. Inspired by the successful synthesis of heavily rare‐earth ion doped ZnSe@ZnS QDs, we investigated potential applications as X‐ray scintillators. Figure 5g shows the X‐ray radio‐luminescence with different exposure time spectra of ZnSe@ZnS:Eu^3+^ QDs, which exhibits two characteristic peaks at 440.0 nm and 610.0 nm corresponding to ZnSe@ZnS QDs’ band‐edge emission and Eu^3+^ ions transitions, respectively. During continuous exposure to X‐ray irradiation, the sample was monitored with radioluminescence measurements taken at regular intervals. ZnSe@ZnS:Eu^3+^ exhibited radiation stability, with luminescence intensity persisting at its initial value for at least 30 min of exposure (Figure 5g), demonstrating excellent radiation stability. A possible mechanism for the X‐ray scintillator properties of ZnSe@ZnS:Eu^3+^ QDs is depicted in Figure 5h. Under high‐energy X‐ray irradiation, the photoelectric effect generates numerous secondary electrons. After rapid thermalization, electron‐hole pairs are injected into both the conduction band (CB) of ZnSe@ZnS and Eu^3+^ 4f energy levels. Due to the small energy gap, electrons in the CB of ZnSe@ZnS QDs can also transfer into the Eu^3+^ 4f states, enabling radio‐luminescence from both the QDs and the Eu^3+^ ions.

To further evaluate the potential of ZnSe@ZnS:Eu^3+^ QDs for X‐ray imaging, QD‐based uniform films were prepared according to the illustrated procedure and shown in Figure 5i. The absorption coefficient of ZnSe@ZnS and ZnSe@ZnS:Eu^3+^ QDs were showed at Figure 5j. Then both ZnSe@ZnS and ZnSe@ZnS:Eu^3+^ QDs films were utilized for X‐ray imaging (Figure 5k–m, and Supplementary Figures S20–S21). The undoped ZnSe@ZnS film exhibited a very weak X‐ray induced spring pattern and was almost invisible under identical irradiation. In contrast, doping the ZnSe@ZnS nanocrystals with Eu^3+^ substantially increases X‐ray absorption, producing a pronounced X‐ray induced spring pattern with a spatial resolution of 5.5 lp/mm, demonstrating successful incorporation of rare‐earth ions into traditional II‐VI quantum dots and signaling potential for clinic, security, and other X‐ray scintillator applications.

## 3 Conclusions

In summary, the present work demonstrates that rare‐earth doped II‐VI QDs engage multiple coupling pathways that exploit the abundant 4f electrons characteristic of rare‐earth elements alongside the strong optical absorption cross‐section of QDs. The approach affords a rich repertoire of rare‐earth dopants and precise control over nanocrystal morphology. Significantly, the strategy exhibits generalizability to other lanthanide ions. Looking forward, the atomic‐level insight into dopant incorporation and energy transfer established herein provides a foundation for further optimization, including increasing the effective doping concentration, engineering heterostructures to boost energy‐transfer efficiency, and exploring hybrid systems that merge QDs’ large absorption cross‐sections with the sharp emissive lines of rare‐earth ions. Moreover, the excellent X‐ray absorption and radioluminescence performance of these rare‐earth doped QDs render them promising candidates for radionuclide quantification and on‐site radioactive detection, such as handheld nuclear identification and field radiation monitoring. We anticipate that the proposed dual hard‐base anchoring paradigm will catalyze the emergence of a new class of rare‐earth doped QDs materials with tailored optical properties, enabling applications ranging from security screening to photovoltaics and quantum information processing.

## 4 Experimental Section

### 4.1 Materials

Oleylamine (OAm, 70%), 1‐octadecene (ODE, 90%), oleic acid (OA, 90%), zinc acetate (ZnAc_2_, 99.99%), zinc chloride (ZnCl_2_, 99%), europium (III) acetate tetrahydrate (EuAc_3_·4H_2_O, 99%), ytterbium (III) acetate tetrahydrate (YbAc_3_·4H_2_O, 99%), cerium (III) acetate tetrahydrate (CeAc_3_·4H_2_O, 99%), praseodymium (III) acetate tetrahydrate (PrAc_3_·4H_2_O, 99%), terbium (III) acetate tetrahydrate (TbAc_3_·4H_2_O, 99%), samarium (III) acetate tetrahydrate (SmAc_3_·4H_2_O, 99%) and 1‐octanehiol (98%) were purchased from Thermo Scientific. Hydroxypropyl acrylate (HPA, 99%), 4, 4'‐thiodiphthalic anhydride (TDPA, 99%), and selenium powder (Se, 99.99%) were purchased from Tokyo Chemical Industry. Tri‐n‐octylphosphine (TOP, 97%), 1,10‐phenanthroline (Phe, 99%), and Poly methyl methacrylate (PMMA, (C_5_H_8_O_2_)_n_) were purchased from Innochem. Toluene (C_7_H_8_, AR) and Cyclohexane (C_6_H_12_, AR) were purchased from Beijing chemical works. All the reagents were used as received without further purification.

### 4.2 Synthesis of 0.25 mol/L Zn(OA)_2_ Precursors

Zinc acetate dihydrate (ZnAc_2_, 1834.8 mg), oleic acid (OA, 16 mL), and 1‐octadecene (ODE, 24 mL) were added to a 100 mL flask at room temperature under a nitrogen atmosphere. The mixture was heated to 120°C and degassed three times to remove water and oxygen. After degassing, nitrogen was reintroduced, and the mixture was maintained at 120°C for 30 min, during which it gradually transformed from an initial white turbidity to a clear yellow solution. Once the reaction was complete, the mixture was cooled to 80°C, and zinc oleate (Zn(OA)_2_) was obtained and transferred for further use. The Zn(OA)_2_ solution should be stored at 4°C for subsequent experiments.

### 4.3 Synthesis of 0.1 mol/L Eu(OA)_3_ Precursors

Europium (III) acetate tetrahydrate (EuAc_3_·4H_2_O, 400.1 mg), oleic acid (OA, 2 mL), oleylamine (OAm, 2 mL) and 1‐octadecene (6 mL) were added to a 50 mL flask at room temperature under a nitrogen atmosphere. The mixture was then heated to 120°C and degassed three times to remove residual water and oxygen. After degassing, nitrogen was reintroduced, and the mixture was maintained at 120°C for 20 min, during which it gradually transformed from a white turbidity to a clear yellow solution. Once the reaction was complete, the mixture was cooled to 60°C, and europium oleate (Eu(OA)_3_) was obtained and transferred for further use. The Eu(OA)_3_ solution should be stored at 4°C for subsequent experiments.

### 4.4 Synthesis of ZnSe@ZnS QDs

A mixture of oleylamine (OAm, 1 mL), oleic acid(OA, 1 mL), 1‐octadecene (ODE, 8 mL), and 1.6 mL Zn(OA)_2_ precursors was added to a 50 mL flask at room temperature under a nitrogen atmosphere [34, 35]. The mixture was then heated to 120°C and degassed three times to remove residual water and oxygen. nitrogen was reintroduced, the mixture was raised to 300°C under nitrogen. Then a rapid injection of 1 mL of Se‐TOP solution, the temperature was maintained for 5 min to allow for core nucleation. The mixture was cooled to 220°C for the addition of 0.2 mL of TOP. For the subsequent shell growth, the temperature was raised to 260°C, and a solution of Zn(OA)_2_ and 1‐octanethiol in ODE was infused 2 h. Following the injection, the reaction was annealed at 295°C for 30 min. Once the reaction was complete, the reaction was cooled to 180°C, then 2 mL toluene was added. After cooling to room temperature, the product was centrifuged with ethanol, washed twice, and finally dissolved in 5 mL cyclohexane for storage.

### 4.5 Synthesis of ZnSe@ZnS:Eu^3+^ QDs

A mixture of oleic acid (OA, 1 mL), 1‐octadecene (ODE, 5 mL), 0.005 mL Eu(OA)_3_ and 0.195 mL Zn(OA)_2_ precursors was added to a 50 mL flask at room temperature under a nitrogen atmosphere [4]. The mixture was stirred at 800 rpm, then purged and degassed three times with nitrogen at room temperature. It was gradually heated to 120°C and maintained for 30 min under nitrogen flow. The system was then switched to a nitrogen atmosphere and heated to 300°C. Then, 1 mL Se‐TOP solution was rapidly injected and the reaction was maintained at 300°C for 5 min before the heater was removed to cool the mixture. At 220°C, 0.2 mL of TOP and 0.4 mL ZnF_2_/ODE (100 mg/ mL) was injected. When temperature was then increased to 240°C, 0.6 mL Zn(OA)_2_ was slowly injected, followed by a 30‐min reaction at 280°C. The temperature was then raised to 310°C. During heating, two solutions were pumped over 2 h: Solution A, Zn(OA)_2_ precursor (5 mL with injection rate 2.5 mL/h), and Solution B, 0.13 M octanethiol in ODE (5 mL with injection rate 2.5 mL/h). The mixture was maintained at 310°C for 30 min. Once the reaction was complete, the reaction was cooled to 180°C, then 2 mL toluene was added. After cooling to room temperature, the product was centrifuged with ethanol, washed twice, and finally dissolved in 5 mL cyclohexane for storage.

### 4.6 Synthesis of (Content of Eu, 2%, 3%, 4%) ZnSe@ZnS:Eu^3+^ QDs

The procedure follows the same steps as the 1% Eu doped QDs: ZnSe@ZnS QDs were synthesized by varying only the Zn(OA)_2_ and Eu(OA)_3_ precursors to modulate Eu content: 2% Eu used 0.19 mL Zn(OA)_2_ and 0.01 mL Eu(OA)_3_, 3% Eu used 0.185 mL Zn(OA)_2_ and 0.015 mL Eu(OA)_3_, and 4% Eu used 0.18 mL Zn(OA)_2_ and 0.02 mL Eu(OA)_3_, with all other steps and reagents identical to the 1% Eu protocol.

### 4.7 Synthesis of (Ln: Yb, Ce, Pr, Tb, Sm) ZnSe@ZnS: Ln QDs

The procedure follows the same steps as the Eu:ZnSe@ZnS QDs synthesis, differing only in the composition of Ln. All other steps and reagent quantities are identical to the ZnSe@ZnS:Eu^3+^ QD synthesis protocol.

### 4.8 Synthesis of OAm‐Zn Ligands

An OAm‐Zn solution (0.1 M) for the chloride treatment was prepared by heating 0.545 g of ZnCl_2_ (4 mmol) in a mixture of OAm (20 mL) and TOP (20 mL) at 150°C for 30 min under vacuum [36].

### 4.9 Synthesis of OAm‐Zn Passivated ZnSe@ZnS:Eu^3+^ QDs

A mixture of 0.1 mL OAm‐Zn and 5 mL cyclohexane solution of Eu:ZnSe@ZnS QDs was added to a 25 mL flask at room temperature under a nitrogen atmosphere. It was gradually heated to 50°C and maintained for 30 min. After cooling to room temperature, the product was centrifuged with ethanol, washed twice, and finally dissolved in 5 mL of chloroform for storage.

### 4.10 Synthesis of Phe/OAm‐Zn Passivated ZnSe@ZnS:Eu^3+^ QDs

A mixture of 0.1 mL chloroform solution of Phe (0.1M) and 5 mL chloroform solution of OAm‐Zn mixted ZnSe@ZnS:Eu^3+^ QDs was added to a 25 mL flask at room temperature under a nitrogen atmosphere. It was gradually heated to 50°C and maintained for 30 min. After cooling to room temperature, the product was centrifuged with ethanol, washed twice, and finally dissolved in 5 mL cyclohexane for storage.

### 4.11 Fabrication of QDs‐PMMA

Briefly, 1.2 g of PMMA was dissolved in 8 mL of toluene and stirred at 50°C for 1 h. Subsequently, the prepared 18.14 mM QDs were added to the PMMA precursor and stirred at room temperature for an additional hour. The resulting mixture (QDs and PMMA colloid) was then poured onto a pre‐fabricated template and dried either through annealing or air drying to produce a thickness of 2 mm QDs@PMMA films.

## Author Contributions

**Jiawang Liu**: data curation. **Yinghui Wang**: software, data curation. **Boyuan Shen**: methodology. **Jiabin Cui**: conceptualization, investigation, funding acquisition, writing – review and editing, writing – original draft. **Haorong Jiao**: methodology. **Jicun Ma**: methodology, data curation, writing – original draft. **Jiada Fan**: methodology, software. **Chenhao Yang**: methodology, validation. **Jialiang Xu**: methodology. **Hui Cai**: methodology, writing – review and editing.

## Conflicts of Interest

The author declares no conflicts of interest.

## Supporting information




**Supporting File**: advs75871‐sup‐0001‐SuppMat.docx.

## Data Availability

The data that support the findings of this study are available from the corresponding author upon reasonable request.
