# Supplementary material for: Rare‐Earth Mediated Engineering in ZnSe@ZnS:Eu3 + to Simultaneously Achieve Structural Modulation and Atomic Eu Doping Sites for X‐Ray Imaging
Source: Adv Sci (Weinh). 2026 May 29:e75871. Online ahead of print. doi: 10.1002/advs.75871 (PMC13336082; doi:10.1002/advs.75871)
Supplement: Supplementary file 1 — Supporting File: advs75871‐sup‐0001‐SuppMat.docx. [file ADVS-9999-e75871-s001.docx]

Supporting Information

**Rare-Earth Mediated Engineering in ZnSe@ZnS:Eu³⁺ to Simultaneously Achieve Structural Modulation and Atomic Eu Doping Sites for X-ray Imaging**

*Jicun Ma^1†^, Chenhao Yang^1†^, Jiada Fan^1†^, Haorong Jiao^1^, Jiawang Liu^2^, Jialiang Xu^1*^, Hui Cai^1*^, Yinghui Wang^2*^, Boyuan Shen^3^, Jiabin Cui^1*^*

^1^ State Key Laboratory of Radiation Medicine and Protection, School of Radiation Medicine and Protection, Collaborative Innovation Center of Radiological Medicine of Jiangsu Higher Education Institutions, Soochow University, Suzhou 215123, China.

E-mails: jlxu@suda.edu.cn, caihui@suda.edu.cn, and jiabin.cui@suda.edu.cn

^2^ Femtosecond Laser Laboratory, College of Physics, Synergetic Extreme Condition High-Pressure Science Center, Jilin University, Changchun, 130012, PR China.

E-mails: yinghui_wang@jlu.edu.cn

^3^ State Key Laboratory of Bioinspired Interfacial Materials Science Institute of Functional Nano & Soft Materials (FUNSOM) Soochow University Suzhou, Jiangsu 215123, P. R. China.

†: The authors have equally contributed to the manuscript.

**Table of Contents**

**Characterization**...................................................................................................................................3

**Supplementary Fig. 1:** Synthesis process of ZnSe@ZnS QDs............................................................4

**Supplementary Fig. 2-12:** TEM, XRD and STEM-EDS mapping analysis........................................5

**Supplementary Fig. 13-15:** TA spectrum analysis and calulation of ZnSe@ZnS..............................16

**Supplementary Fig. 16:** Photoluminescence lifetime decay of ZnSe@ZnS:Eu^3+^ QDs......................19

**Supplementary Fig. 17-18:** TEM and characterization of ZnSe@ZnS with different Ln(III) ions…20

**Supplementary Fig. 19**: TA analysis of ZnSe@ZnS doped with different Ln(III) ions…………….22

**Supplementary Fig. 20-21**: The X-ray imaging of LYSO and ZnSe@ZnS QDs...............................23

**Supplementary Table 1**: The different configuration ZnSe@ZnS:Eu^3+^............................................25

**Supplementary Table 2-4**: The summary of calculation results........................................................26

**Supplementary Table 5**: The PLQY of the QDs...............................................................................29

**Characterization**

The UV-vis-NIR absorption spectrum was measured using a Lambda 35 spectrophotometer. Photoluminescence and radioluminescence spectra were recorded with a QE Pro fluorescence spectrophotometer (Ocean Insight), equipped with a miniature X-ray source (AMPEK, mini-X2). X-ray induced luminescence images were captured using a digital camera (Work Power WP-GS1200). Timeresolved photoluminescence measurements were conducted with a FL3-TCSPC system (HORIBA JOBIN YVON). Transient absorption spectroscopy measurements were conducted with a ultrafast transient absorption fluorescence microspectroscopy system (PH-Tuning + TA100). Transmission electron microscopy (TEM) and high-resolution TEM (HRTEM) analyses were performed with a Talos F200S G2 microscope operated at 200 kV. High-resolution scanning transmission electron microscopy (STEM) imaging and elemental mapping were carried out using a Themis Z aberration-corrected STEM (Thermo Fisher Scientific) at 300 kV, equipped with a high angular annular dark field (HAADF) detector for STEM imaging and a Super-X energy dispersive X-ray spectroscopy (EDS) detector for elemental analysis. X-ray powder diffraction (XRD) measurements were performed with a D8 Advance diffractometer.

**TA Spectral Measurement**

A femtosecond transient absorption (TA) spectroscopy is designed as below. The primary light source was a tunable femtosecond laser (PHAROS, Light Conversion) with a repetition rate of 100 kHz, a pulse energy of 200 μJ, and a pulse duration of 200 fs. The output beam was divided into pump and probe paths. The former enters into optical parametric amplifier (ORPHEUS, Light Conversion) and the output laser is used to excite the sample, whose wavelength can be tuned from 350 to 2000 nm. The probe beam was generated by focusing both the fundamental 1030 nm beam and its frequency-doubled 515 nm component into a sapphire crystal, yielding a white-light continuum covering 380-1000 nm. A motorized translation stage provided precise temporal delay of the pump beam with a time resolution of 14 fs, and the pump beam was modulated at 250 Hz using an optical chopper (C995, Terahertz Technologies). Both beams were spatially and temporally overlapped at the sample position via a common reflector. Femtosecond TA spectra were acquired using a high-sensitivity spectrometer (Avantes AvaSpec-ULS2048×16).

**Supplementary Figures and Tables**


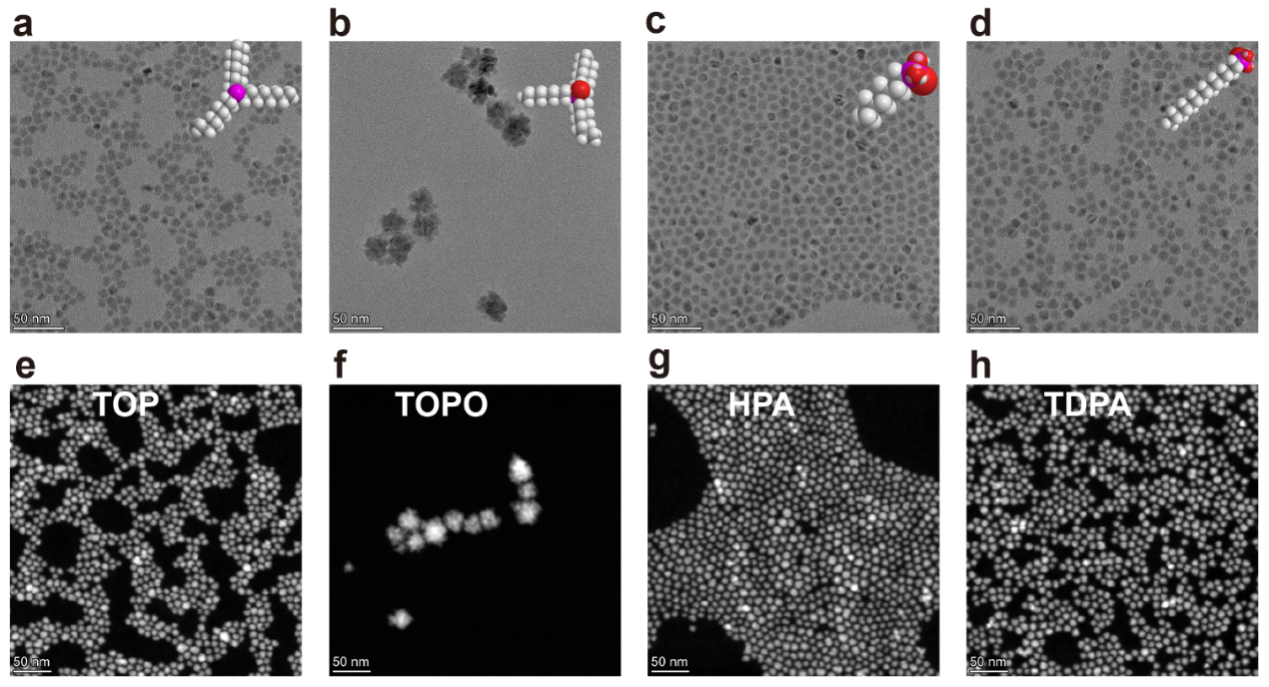


**Supplementary Figure 1.** **Effect of surface ligands on the morphology of ZnSe@ZnS QDs.** TEM images of QDs capped with TOP (a), TOPO (b), HPA (c), and TDPA (d). Scale bar: 50 nm.


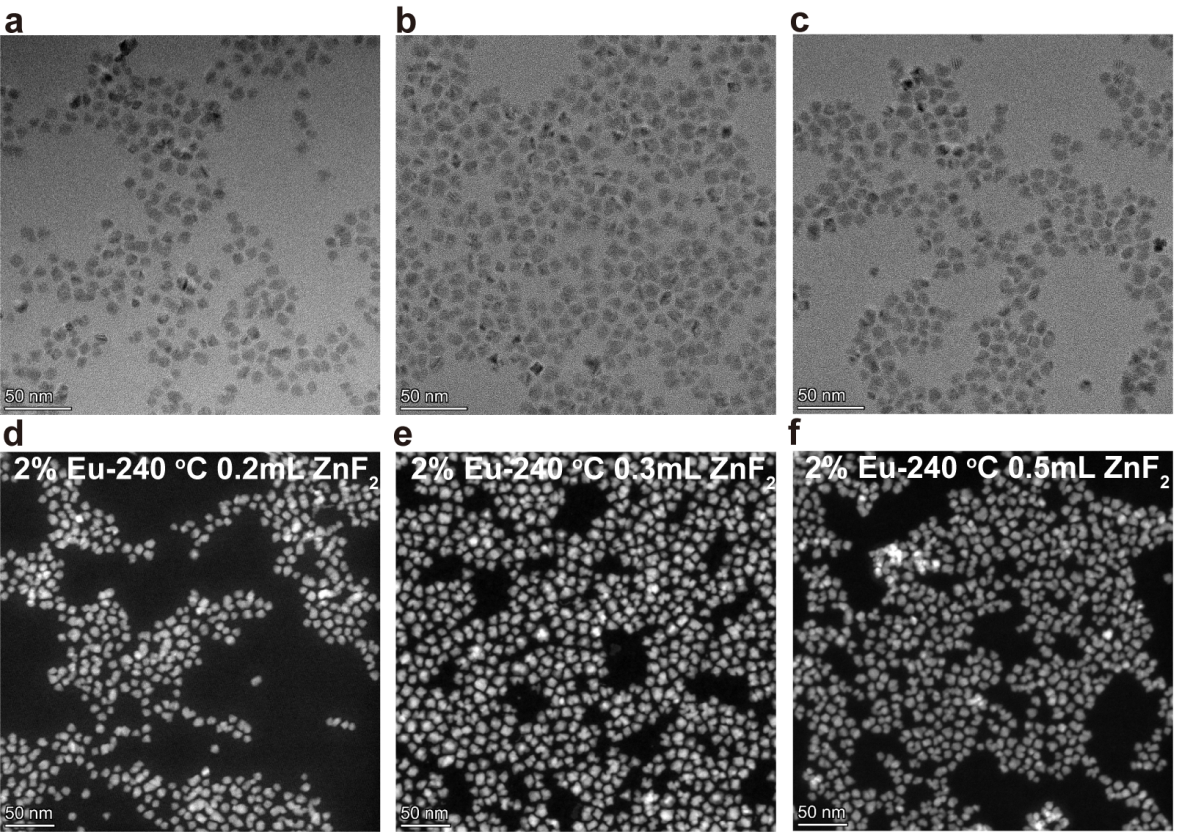


**Supplementary Figure 2.** **Morphology of 2%** **ZnSe@ZnS:Eu^3+^ QDs synthesized with different amounts of ZnF_2_.** TEM (a-c) and corresponding STEM images (d-f) of QDs prepared with ZnF₂ volumes of 0.2 mL (a, d), 0.3 mL (b, e) and 0.5 mL (c, f), respectively. Scale bar: 50 nm.


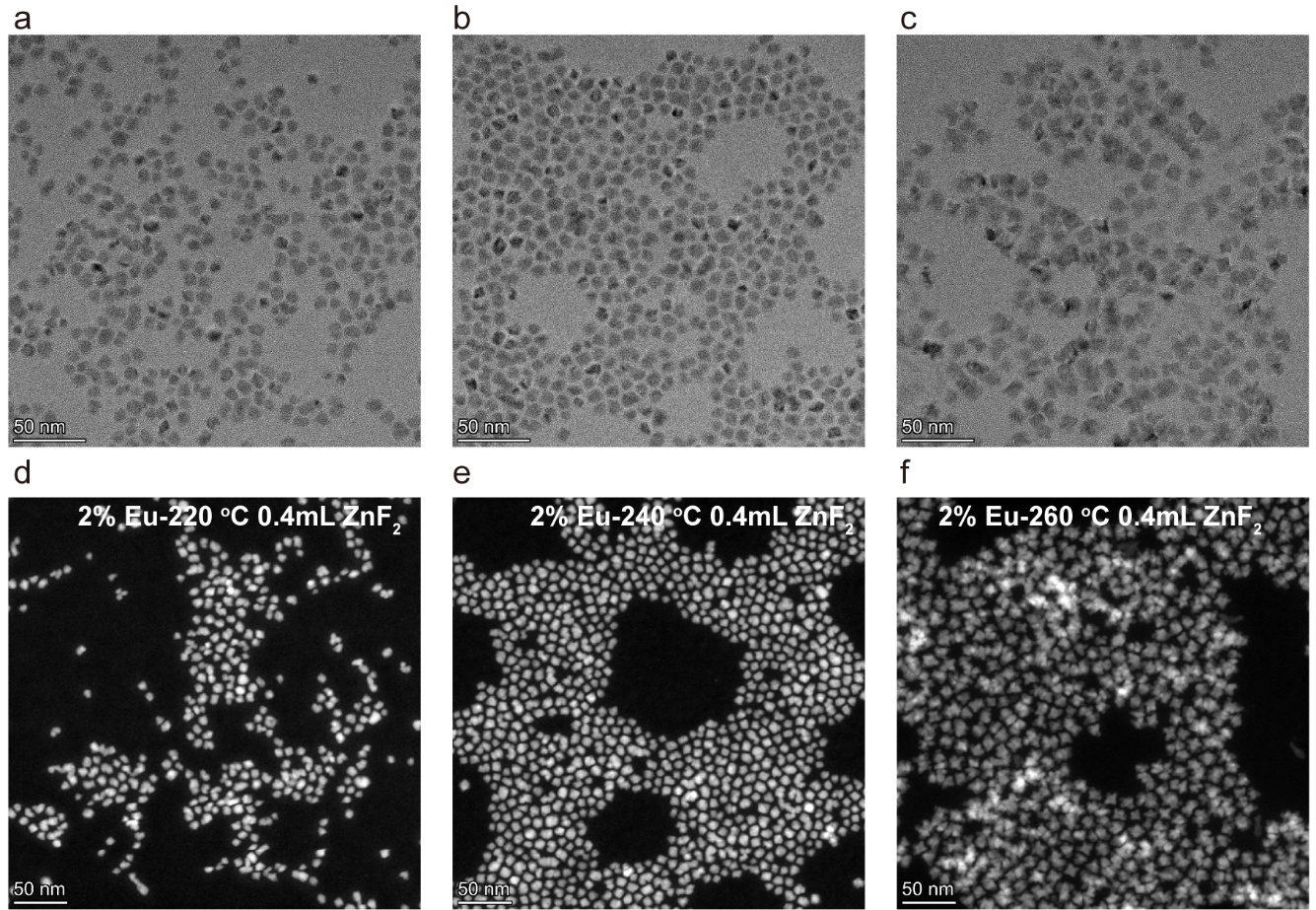


**Supplementary Figure 3. Morphology of ZnSe@ZnS:Eu^3+^ QDs synthesized at different Eu(III) ion injection temperatures.** TEM (a-c) and corresponding STEM images (d-f) of QDs prepared at temperatures of 220 °C (a, d), 240 °C (b, e), and 260 °C (c, f), respectively. Scale bar: 50 nm.


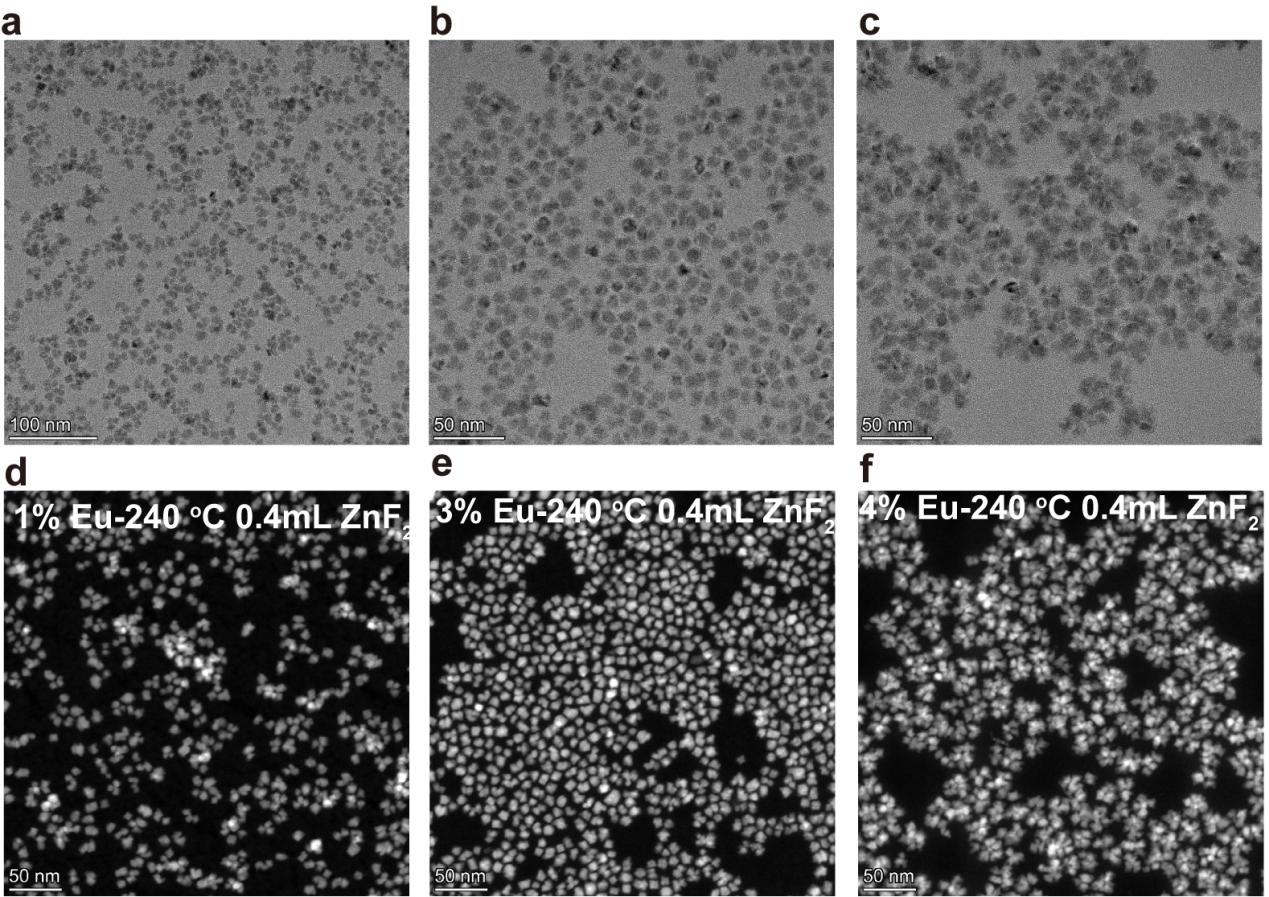


**Supplementary Figure 4.** **Morphology and structure of ZnSe@ZnS:Eu^3+^ QDs with varying Eu(III) ion content.** TEM (a-c) and corresponding STEM (d-f) images of the QDs with a nominal Eu(III) doping concentration of 1% (a, d), 2% (b, e) and 4% (c, f). Scale bar: 50 nm.


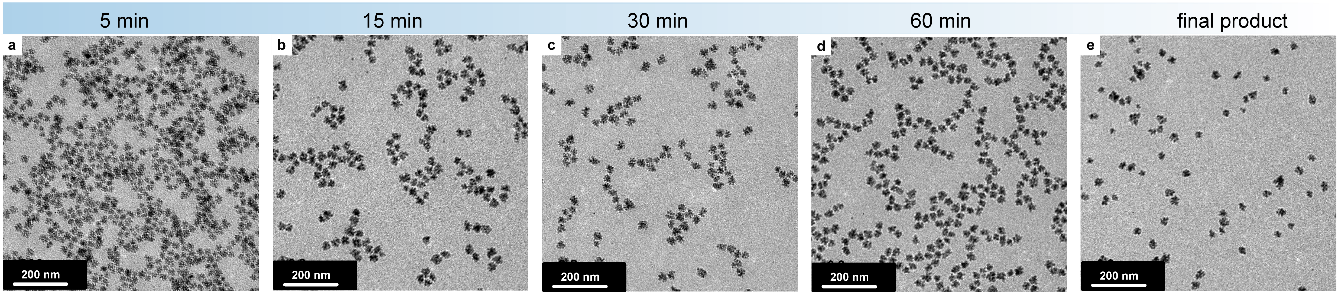


**Supplementary Figure 5. Morphology and structure of ZnSe@ZnS:Eu^3+^ QDs with different reaction times.** TEM images of the QDs of 5 min,15 min, 30 min, 60 min and final product. Scale bar: 200 nm.


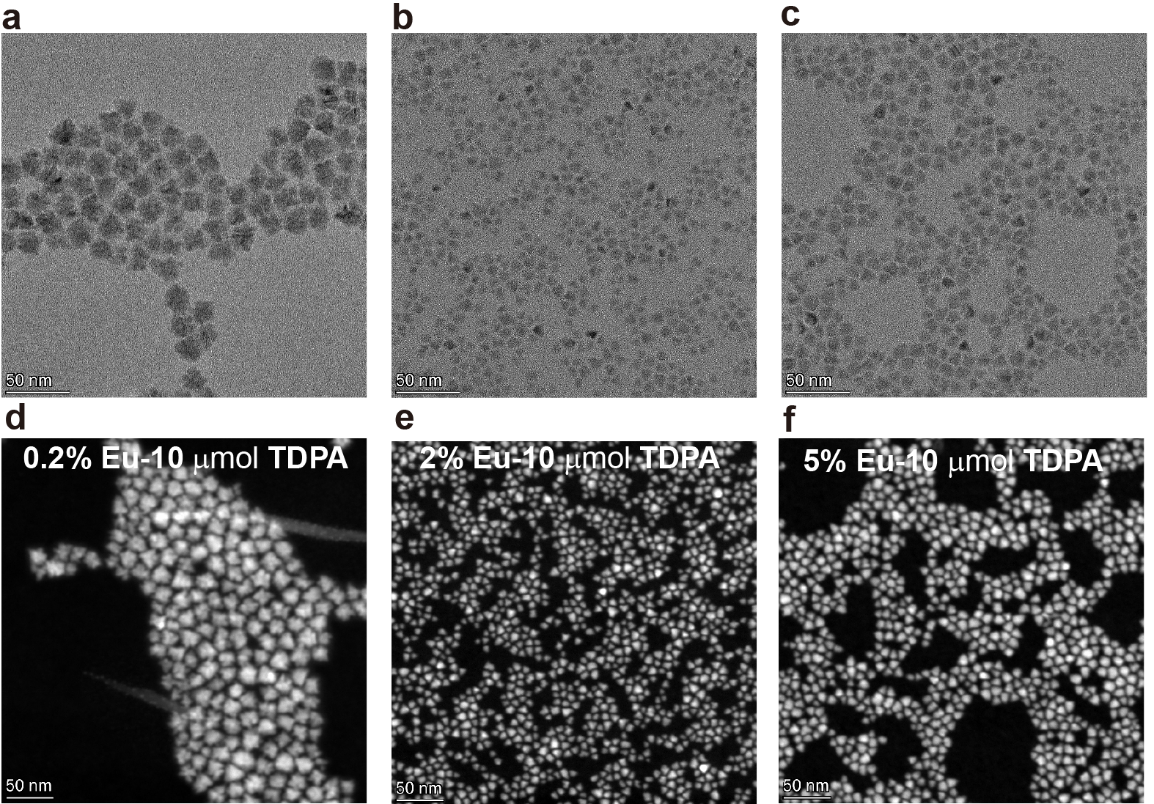


**Supplementary Figure 6. Morphology and structure of ZnSe@ZnS:Eu^3+^ QDs with varying Eu(III) ion content.** TEM (a-c) and corresponding STEM (d-f) images of the QDs of 0.2% Eu-10 μmol TDPA (a, d), 2% Eu-10 μmol TDPA (b, e), and 5% Eu-10 μmol TDPA (c, f). Scale bar: 50 nm.


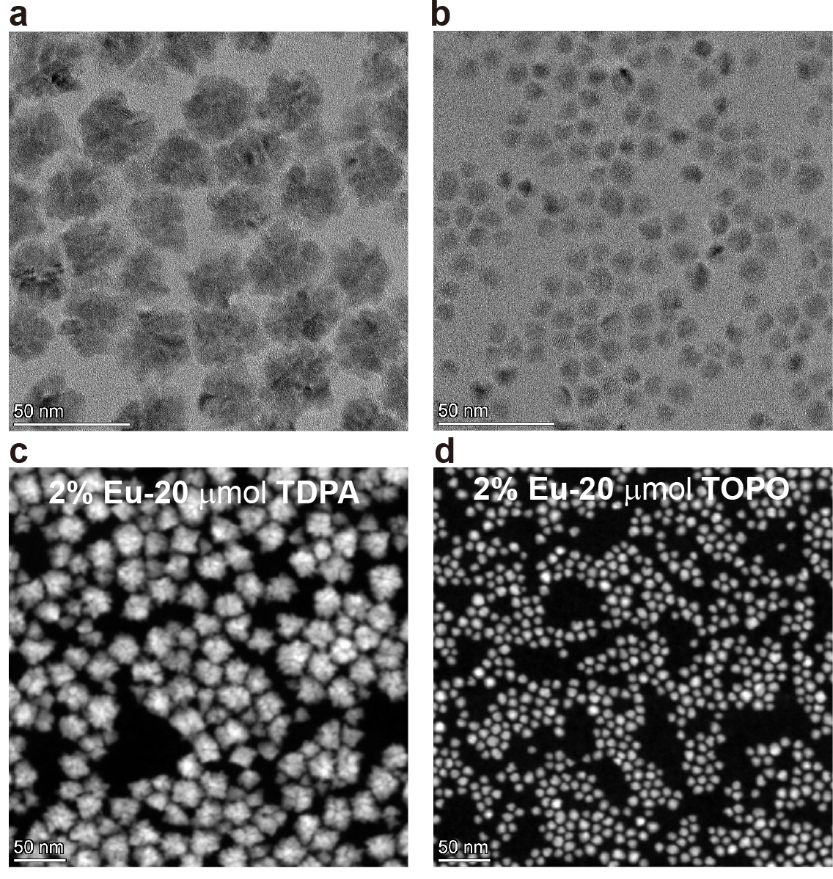


**Supplementary Figure 7. Effect of surface ligands on the morphology of ZnSe@ZnS:Eu^3+^ QDs.** TEM (a-b) and STEM (c-d) images of QDs capped with (a,c) TDPA and (b,d) TOPO. Scale bar: 50 nm.


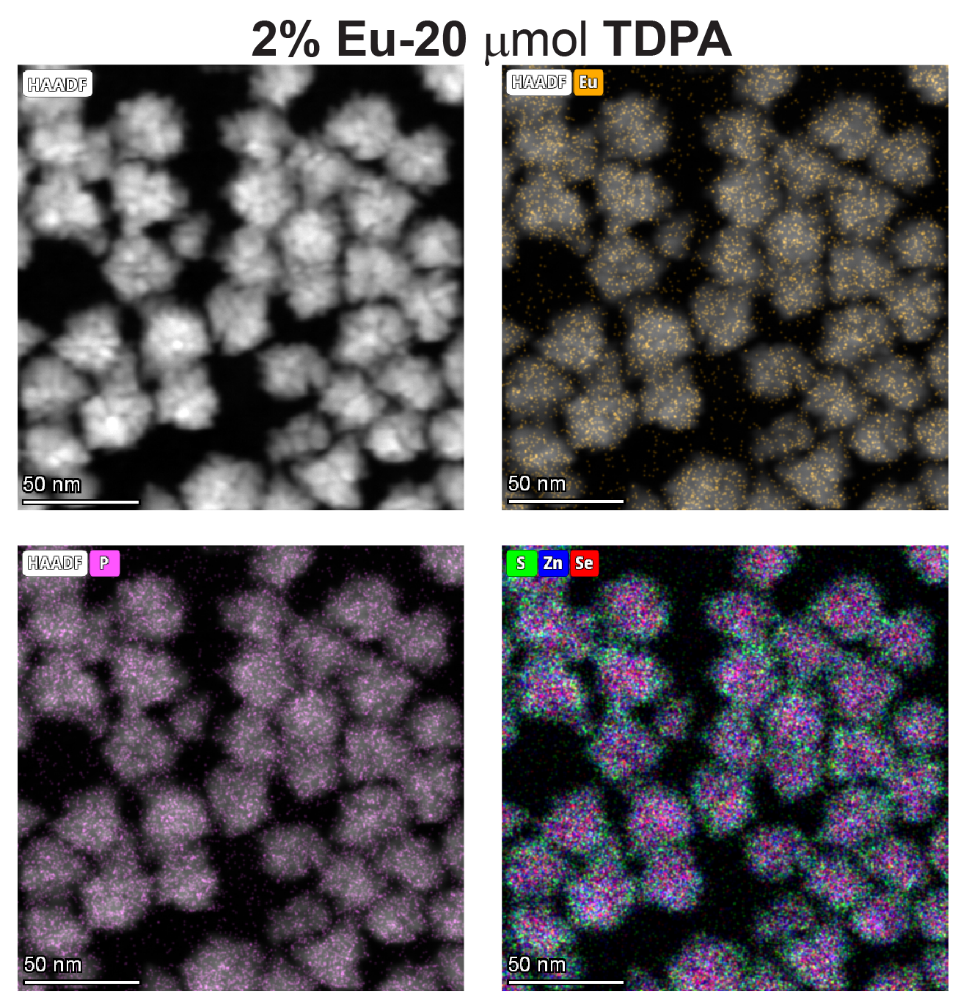


**Supplementary Figure 8. Elemental mapping (Zn, Se, S, Eu, P) of TDPA-passivated ZnSe@ZnS:Eu^3+^ QDs.** Scale bar: 50 nm.


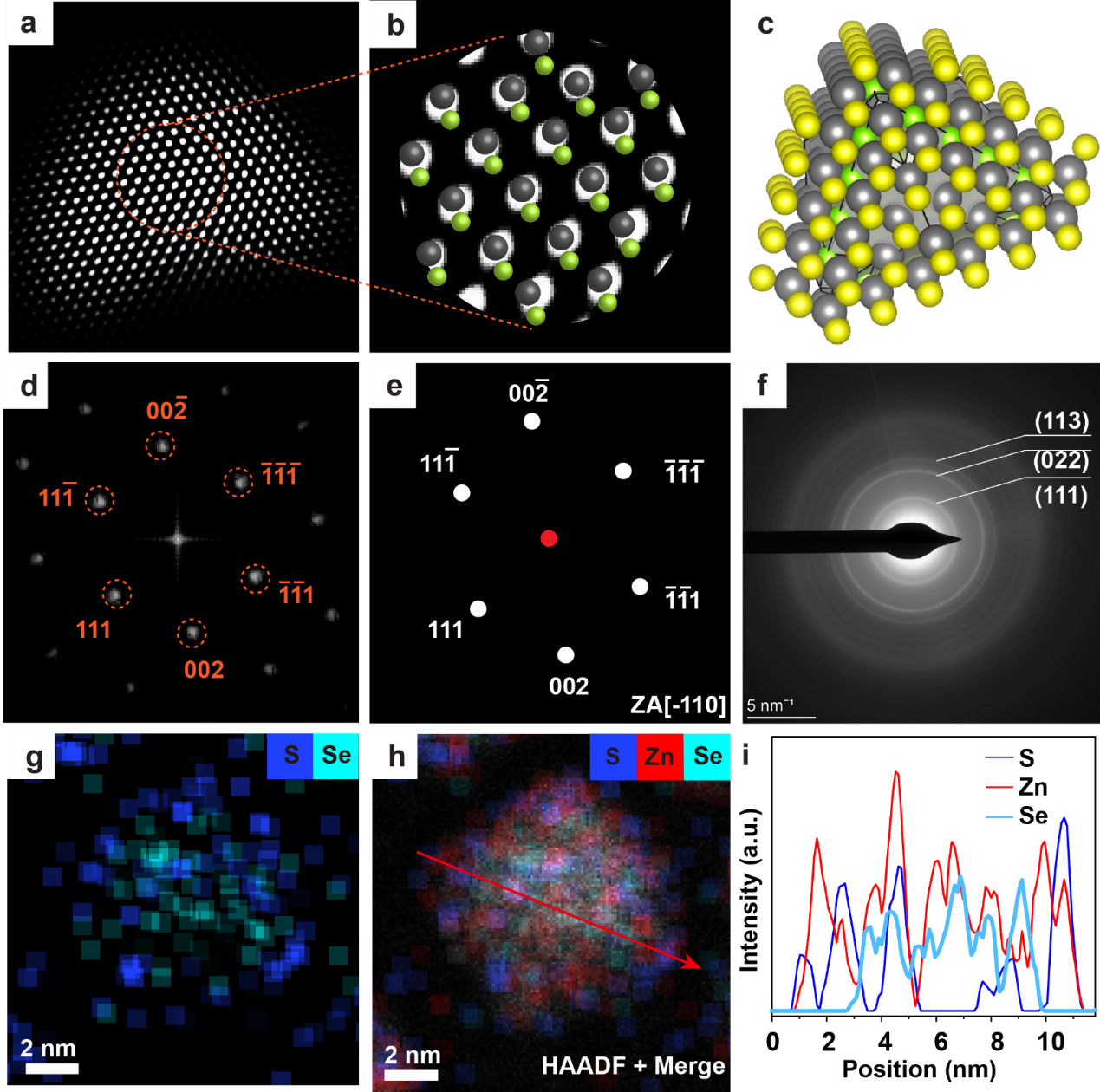


**Supplementary Figure 9.** **Comprehensive structural analysis of ZnSe@ZnS QDs.** (a) STEM overview image. (b-f) High-resolution analysis of a single QD: high-magnification image (b), FFT-reconstruction (c), experimental (d) and theoretical FFT patterns along the 1-axis (ZA) (e), and scattered diffraction pattern (f). (g-i) Elemental composition: EDS mapping (g,h) and line scan (i).


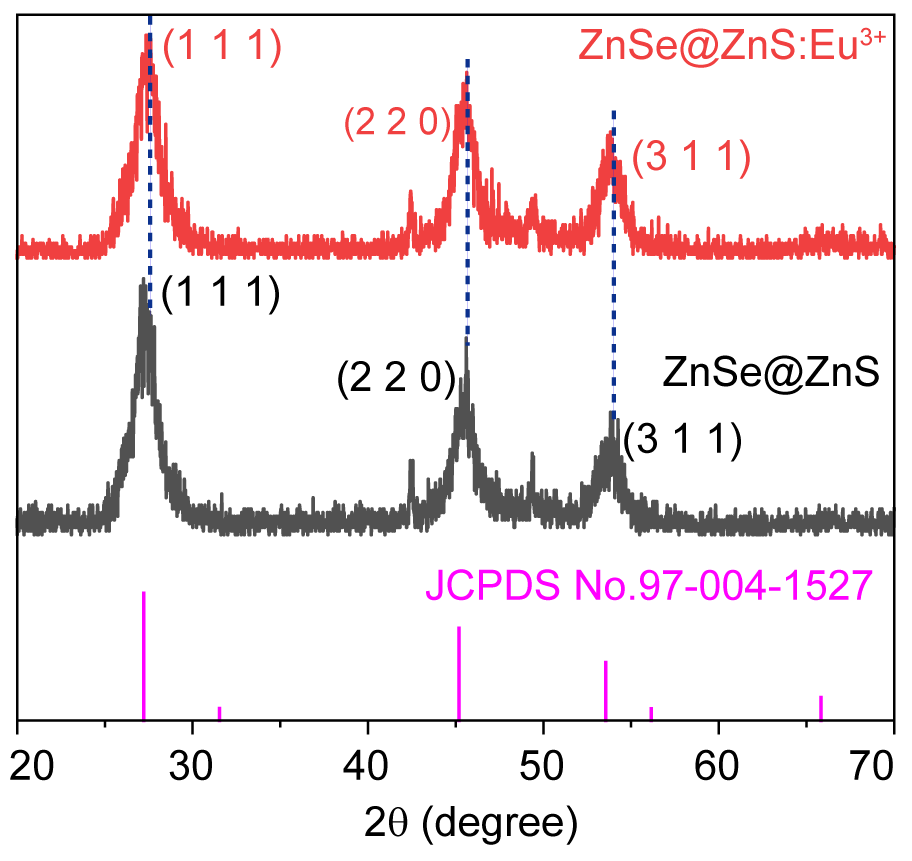


**Supplementary Figure 10**. **X‑ray diffraction (XRD) patterns.** The XRD patterns of undoped ZnSe@ZnS and ZnSe@ZnS:Eu³⁺ QDs.


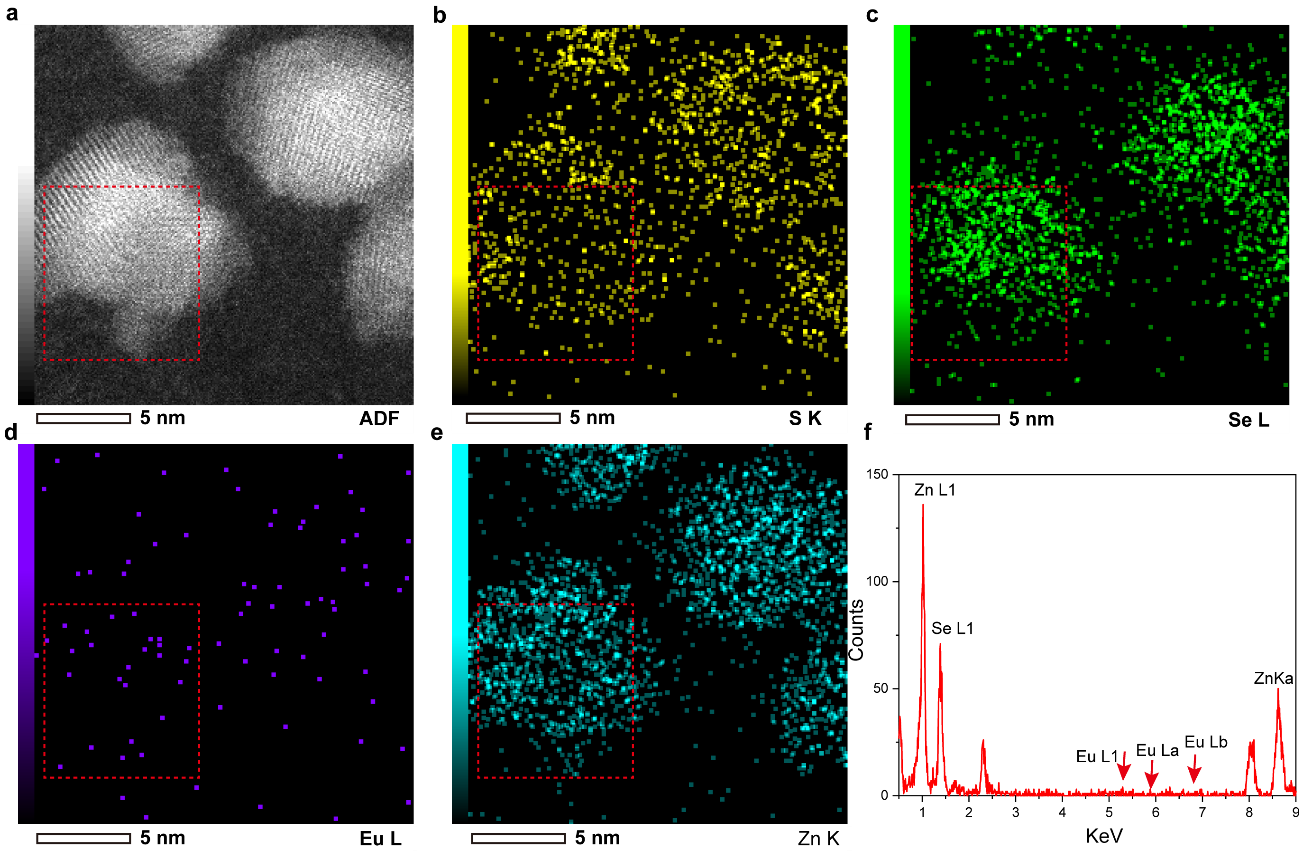


**Supplementary Figure 11**. **Structural analysis of tetragonal tip growth ZnSe@ZnS:Eu^3+^ QDs.** HAADF-STEM image (a) STEM-EDS elemental mapping (b-e), and EDS spectrum (f).


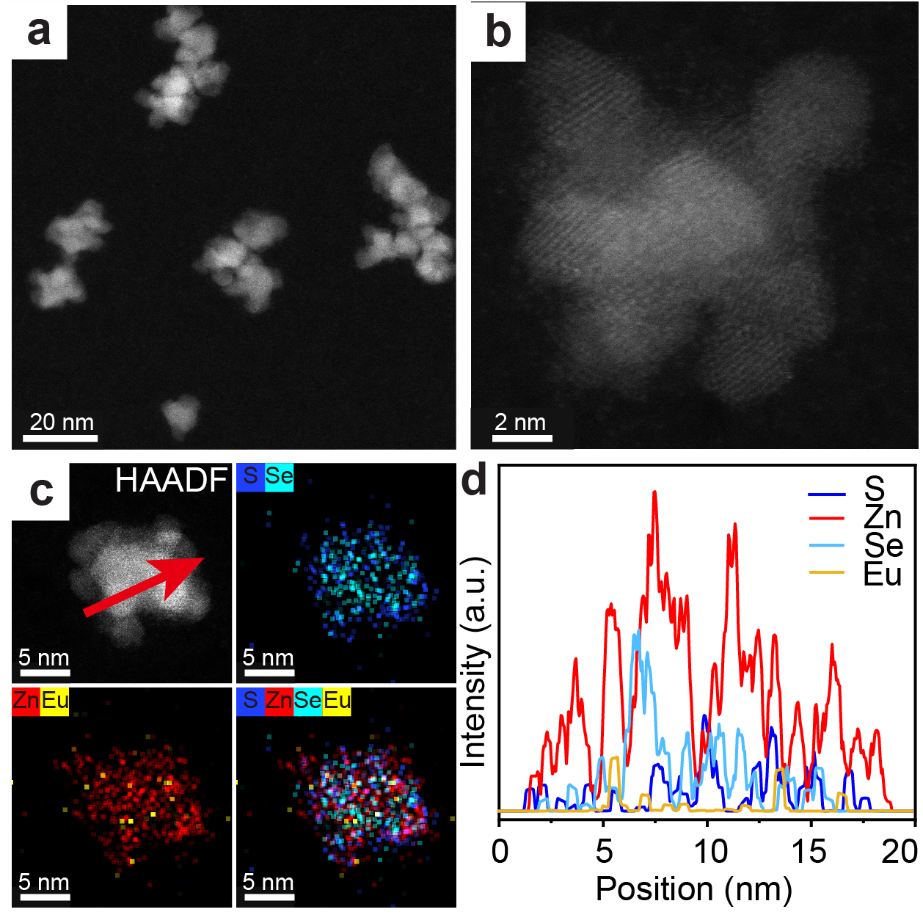


**Supplementary Figure 12.** **Structural analysis of tetragonal island growth ZnSe@ZnS:Eu^3+^ QDs.** HAADF-STEM image (a-b), STEM-EDS elemental mapping (c), and EDS line scan profile (d).


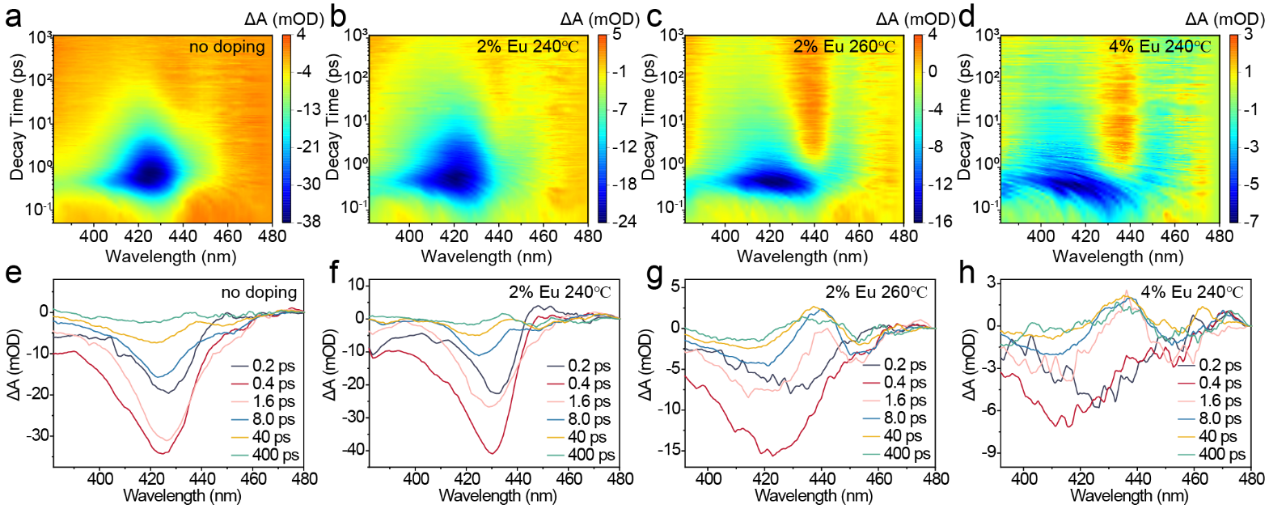


**Supplementary Figure 13. Transient absorption (TA) spectroscopy of ZnSe@ZnS:Eu^3+^ QDs synthesized at different Eu(III) ion injection temperatures.** Two-dimensional TA spectra (a-d) and corresponding TA kinetics probed at specific wavelengths (e-h) for no doping (a, e), 2%, 240 °C (b, f), 2%, 260 °C (c, g) and 4%, 240 °C (d, h), measured under 365 nm excitation.


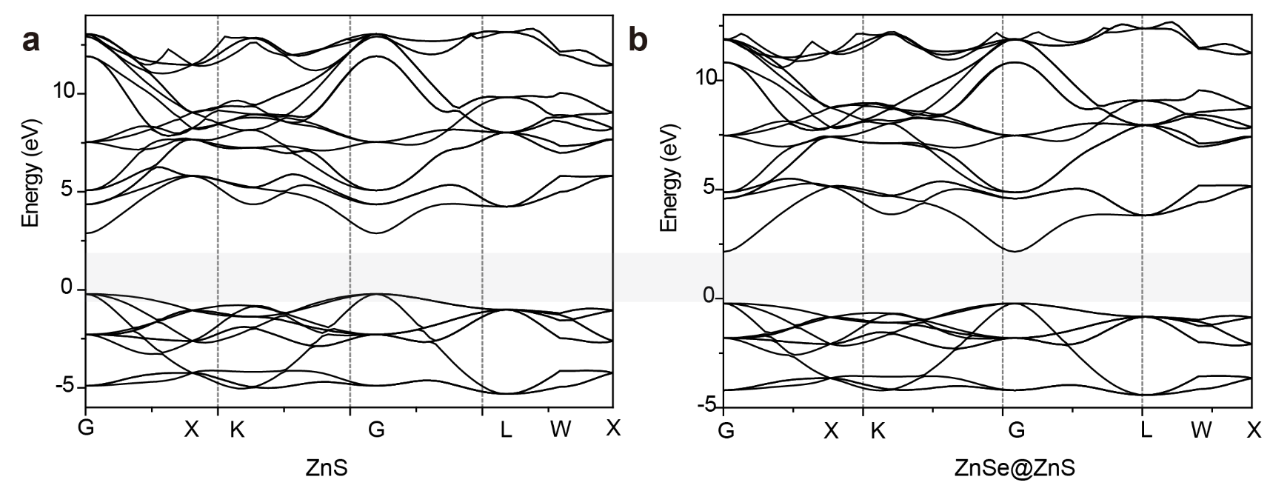


**Supplementary Figure 14.** **Brillouin zone of QDs.** Brillouin zone of ZnS QDs (a) and ZnSe@ZnS QDs (b).


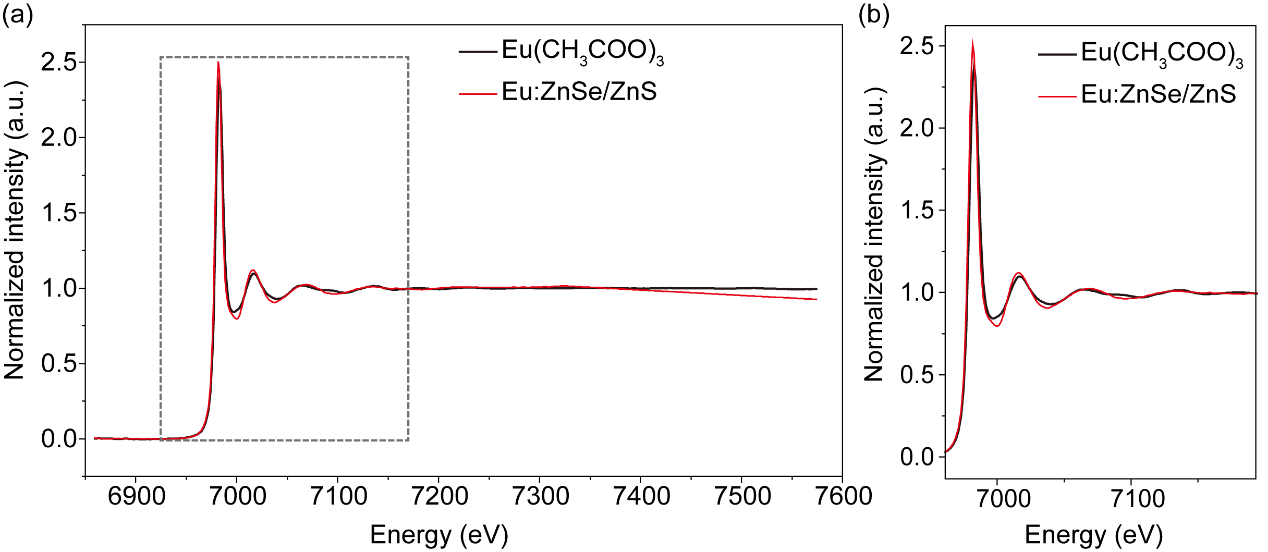


**Supplementary Figure 15.** **XANES spectra of Eu(CH_3_COO)_3_ and ZnSe@ZnS:Eu^3+^ QDs.** Full-range spectra (a) and expanded view (b) of Eu(CH_3_COO)_3_ and Eu:ZnSe@ZnS QDs.

**
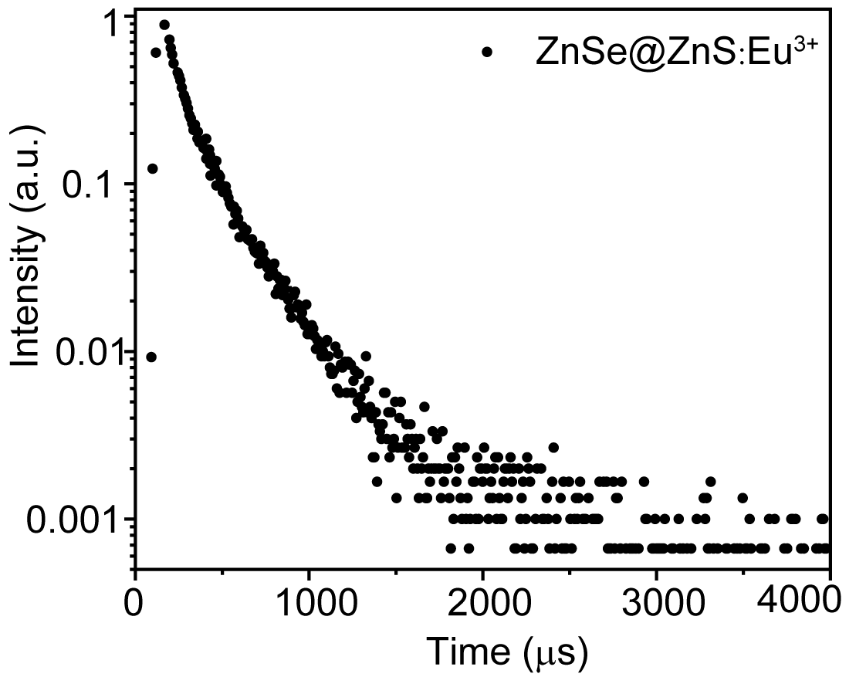
**

**Supplementary Figure 16. The photoluminescence lifetime decay curves under 610 nm of related ZnSe@ZnS:Eu^3+^ QDs.**


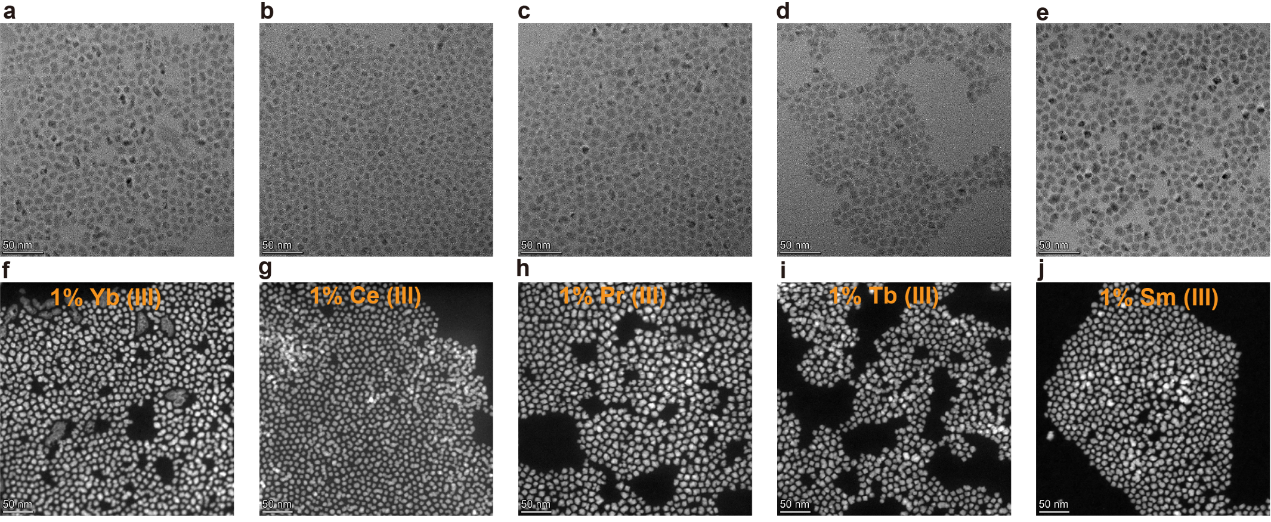


**Supplementary Figure 17. Morphology of** **ZnSe@ZnS:Ln^3+^ QDs incorporating different lanthanide (Ln(III)) ions.** TEM (a-e) and corresponding STEM images (f-j) of QDs doped with 1% Yb(III) (a, f), 1% Ce(III) (b, g), 1% Pr(III) (c, h), 1% Tb(III) (d, i), and 1% Sm(III) (e, j).


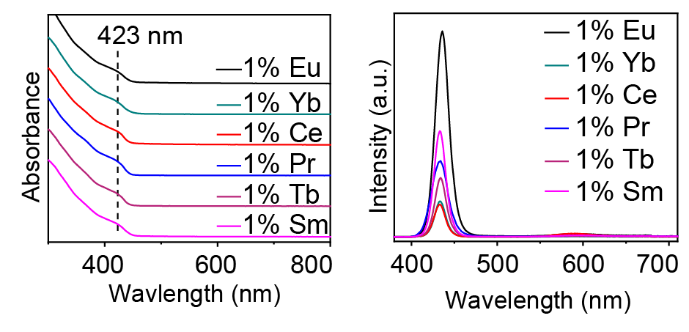


**Supplementary Figure 18. Optical properties of ZnSe@ZnS:Ln^3+^ QDs incorporating different lanthanide (Ln(III)) ions.** UV-Vis absorption (a) and photoluminescence (PL) emission spectra (b) of the respective QD samples.


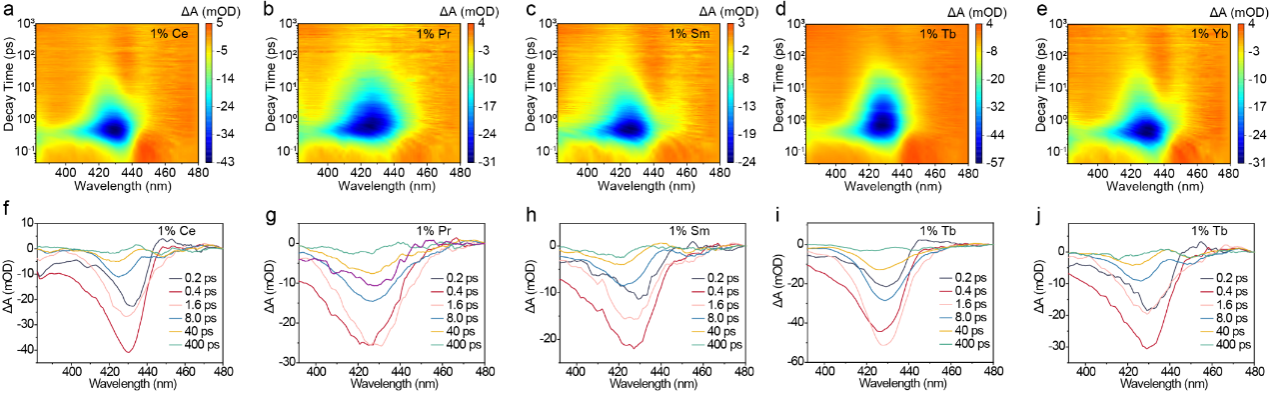


**Supplementary Figure 19.** **Transient absorption (TA) spectroscopy of ZnSe@ZnS QDs doped with different Ln(III) ions.** Two-dimensional TA spectra (a-e) and corresponding TA kinetics probed at specific wavelengths (f-j) for 1% Ce(III) (a, f), 1% Pr(III) (b, g), 1% Sm(III) (c, h), 1% Yb(III) (d, i) and 1% Tb(III) (e, j) doped QDs, measured under 365 nm excitation.

**Supplementary Figures 19a-e** offers the TA map of QDs doped with Ce^3+^, Pr^3+^, Sm^3+^, Tb^3+^ and Yb^3+^ ions, respectively. The spectral feature located at 430 nm was assigned to the bleaching signal, which is much similar to that of pure QDs. After the QDs is doped with rare earth ion, a small negative feature may appear in the TA map of QDs doped with rare-earth ions as seen in **Supplementary Figures 19f-j**. This suggests that the rare earth ions can easily create trapping-sites and cause the simulate emission, in comparison with that of pure QDs.


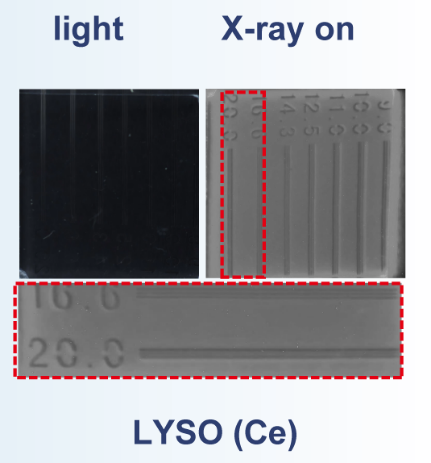


**Supplementary Figure 20**. P**hotograph of the standard X-ray resolution line alignment card.**  The photograph of the standard X-ray resolution line alignment card of LYSO .

**
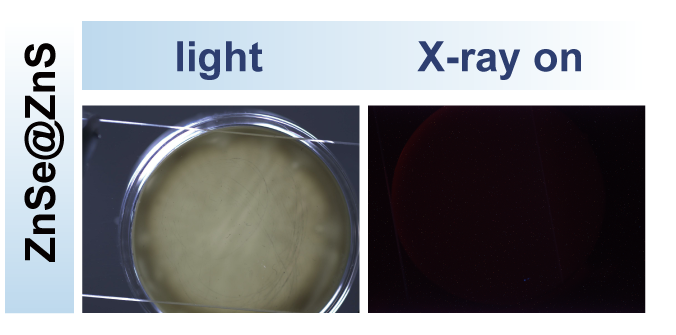
**

**Supplementary Figure 21**. **X-ray imaging.** The film fabrication for X-ray imaging and related X-ray imaging of ZnSe@ZnS QDs.

**Supplementary Table 1. Statistics results for the different configuration ZnSe@ZnS.**

| Frequency (%) | Tetragonal  growth | Tip  growth | Island  growth | Coupled flower  growth |
| --- | --- | --- | --- | --- |
| Tetragonal growth | **98.1** | 28.1 | 14.8 | 0 |
| Tip growth | 1.9 | **65.2** | 4.5 | 0 |
| Island growth | 0 | 6.7 | **62.2** | 2.0 |
| Coupled flower growth | 0 | 0 | 18.5 | **98.0** |

**Supplementary Table 2.** The calculation of adsorption energy between different molecule of ligand and (1 1 1) crystal facets of ZnSe@ZnS QDs

| Molecule | HPA | TDPA | OA | OAm |
| --- | --- | --- | --- | --- |
| E_ads_ (eV) | -1.170 | -1.279 | -0.958 | -1.705 |
| Zn-N/O (Å) | 2.108 | 2.107 | 2.113 | 2.105 |

**Supplementary Table 3.** The calculation of adsorption energy between different crystal facets of ZnSe@ZnS QDs and Fluoride ion.

| Crystal Facet | (111) | (002) | (220) |
| --- | --- | --- | --- |
| E_ads_ (eV) | -5.561 | -6.092 | -2.723 |

**Supplementary Table 4.** EXAFS fitting parameters at the Eu L_3_-edge for various samples (set S_0_^2^ = 0.9).

|  | shell | CN*^a^* | R*^b^*(Å) | σ^2^*^c^*(Å^2^) | ΔE_0_*^d^*(eV) | R factor |
| --- | --- | --- | --- | --- | --- | --- |
| Eu(CH₃COO)₃ | Eu-O | 6* | 1.99±0.01 | 0.0161 | 1.6±1.7 | 0.014 |
| ZnSe@ZnS:Eu^3+^ | Eu-O | 4.33±0.4 | 1.99±0.08 | 0.0038 | -2.6±0.2 | 0.008 |
|  | Eu-F | 2.35±0.1 | 2.31±0.09 | 0.0160 |  |  |
|  | Eu-Eu | 1.05±0.2 | 3.76±0.04 | 0.0105 |  |  |

*^a^CN*: coordination numbers; *^b^R*: bond distance; *^c^σ*^2^: Debye-Waller factors; *^d^*Δ*E*_0_: the inner potential correction. R factor: goodness of fit. Error bounds that characterize the structural parameters obtained by EXAFS spectroscopy were estimated as CN±20%.

**Supplementary Table 5**. The PLQY of the designed QDs.

|  | ZnSe@ZnS | ZnSe@ZnS:Eu^3+^ | ZnSe@ZnS:Eu^3+^+OAm/Phe |
| --- | --- | --- | --- |
| PLQY (%) | 13.5 | 3.7 | 23.1 |
